# Supplementary material for: The symmetry of the Kepler problem, the inverse Ligon-Schaaf mapping and the Birkhoff conjecture
Source: PLoS One. 2018 Sep 13;13(9):e0203821. doi: 10.1371/journal.pone.0203821 (PMC6136791; doi:10.1371/journal.pone.0203821)
Supplement: S1 Document — This document contains proofs and other detailed calculations. (PDF) [file pone.0203821.s001.pdf]

Supporting information to The symmetry of the Kepler problem inverse Ligon-Schaaf mapping and the Birkhoff conjecture

Thomas Sumner Ligon[1*]
[1] Faculty of Physics and Center for NanoScience (CeNS), Ludwig-Maximilians-Universität, München, Germany
* Corresponding author
Email: Thomas.Ligon@physik.uni-muenchen.de (TL)

Abstract

This supporting information consists primarily of detailed calculations for the main paper.

Negative Energy

Notation

Here is a comparison of our notation (on the left) with that of Cushman and Bates[1] (on the right).

The (forward) LS mapping:

$$F = F_2 \circ F_1 \Leftrightarrow \Phi_{LS} = L \circ S$$

The coordinates on $T^*\mathbb{R}^3$:

$$\begin{pmatrix} q \\ p \end{pmatrix} \Leftrightarrow \begin{pmatrix} q \\ p \end{pmatrix}$$

The coordinates on $T^*\mathbb{R}^4$:

$$\begin{pmatrix} r_0 \\ \boldsymbol{r} \\ s_0 \\ \boldsymbol{s} \end{pmatrix} \Leftrightarrow \begin{pmatrix} u \\ u_4 \\ v \\ v_4 \end{pmatrix}$$

The coordinates on T^*S^3:

$$\begin{pmatrix} \xi_0 \\ \boldsymbol{\xi} \\ \eta_0 \\ \boldsymbol{\eta} \end{pmatrix} \Leftrightarrow \begin{pmatrix} r \\ r_4 \\ s \\ s_4 \end{pmatrix}$$

The position vector:

$$\boldsymbol{q} \Leftrightarrow q$$

The absolute value of a vector

$$q \Leftrightarrow \|q\|$$

The inner product:

$$\boldsymbol{q} \cdot \boldsymbol{p} \Leftrightarrow \langle q, p \rangle$$

The rescaling factor:

$$\frac{1}{\sqrt{-2H}} \Leftrightarrow \nu = \frac{1}{\sqrt{-2H}}$$

The angle used in the mapping:

$$\varphi = \sqrt{-2H}\boldsymbol{q}\cdot\boldsymbol{p} \Leftrightarrow v_4 = -\nu^{-1}\langle q,p\rangle$$

Mapping $\boldsymbol{F} = \boldsymbol{F_2} \circ \boldsymbol{F_1}$ in Cushman and Bates notation

Definition S1. *For negative energy and $q \neq 0$, the forward LS mapping is defined by*
(i) Mapping Φ_{LS}

$$\nu = \left(\frac{2}{\|q\|} - \|p\|^2\right)^{-1/2} \tag{s1}$$

$$v_4 = -\nu^{-1}\langle q,p\rangle \tag{s2}$$

$$r = \nu^{-1}\|q\|pcos(v_4) + \left(-\frac{q}{\|q\|} + \langle q,p\rangle p\right) sin(v_4) \tag{s3}$$

$$r_4 = \ (\langle p,p\rangle q - 1)cos(v_4) + v_4 sin(v_4) \tag{s4}$$

$$s = -\nu\nu^{-1}\|q\|psin(v_4) + \nu\left(-\frac{q}{\|q\|} + \langle q,p\rangle p\right) cos(v_4) \tag{s5}$$

$$s_4 = \ -\nu(\langle p,p\rangle q - 1)sin(v_4) - \langle q,p\rangle(v_4) \tag{s6}$$

(ii) Mapping S

$$u = \nu^{-1}\|q\|p \tag{s7}$$

$$u_4 = \langle p,p\rangle q - 1 \tag{s8}$$

$$v = -\frac{q}{\|q\|} + \langle q,p\rangle p \tag{s9}$$

$$v_4 = -\nu^{-1}\langle q,p\rangle \tag{s10}$$

Note 1: Cushman and Bates[1] has a typographical error, writing $v = -\|\mathrm{q}\|\mathrm{q} + \langle \mathrm{q},\mathrm{p}\rangle\mathrm{p}$ instead of $v = -\frac{q}{\|q\|} + \langle q,p\rangle p$.
Note 2: $v_4 = -\varphi$, so
$sin(v_4) = sin(-\varphi) = -sin(\varphi)$,
$cos(v_4) = cos(-\varphi) = cos(\varphi)$.
(iii) Mapping L

$$\nu = \left(\frac{2}{\|q\|} - \|p\|^2\right)^{-1/2} \tag{s11a}$$

$$v_4 = -\nu^{-1}\langle q,p\rangle \tag{s11b}$$

$$L = \begin{pmatrix} cos(v_4) & sin(v_4) \\ -\nu sin(v_4) & \nu cos(v_4) \end{pmatrix} \tag{s12}$$

$$r = ucos(v_4) + vsin(v_4) \tag{s13}$$

$$r_4 = \ u_4 cos(v_4) + v_4 sin(v_4) \tag{s14}$$

$$s = -\nu usin(v_4) + \nu vcos(v_4) \tag{s15}$$

$$s_4 = \ -\nu u_4 sin(v_4) + \nu v_4 cos(v_4) \tag{s16}$$

Proposition S1. *$\Phi_{LS} = L \circ S$.*
Proof. This is a simple substitution. ■

Definition S2. *For negative energy and $q \neq 0$, the inverse LS mapping is defined by*
(i) Mapping ${\Phi_{LS}}^{-1}$

$$v_4 = r_4 sin(v_4) + \frac{s_4}{\sqrt{\langle s,s\rangle}} cos(v_4) \tag{s17}$$

$$q = \langle s,s\rangle\left[-r\left(\frac{s_4}{\sqrt{\langle s,s\rangle}} + sin(v_4)\right) + \frac{s}{\sqrt{\langle s,s\rangle}}\left(r_4 - cos(v_4)\right)\right] \tag{s18}$$

$$p = \frac{rcos(v_4) - \frac{s}{\sqrt{\langle s,s \rangle}} sin(v_4)}{\sqrt{\langle s,s \rangle}\left(1 - r_4 cos(v_4) + \frac{s_4}{\sqrt{\langle s,s \rangle}} sin(v_4)\right)} \tag{s19}$$

(ii) Mapping S^{-1}

$$q = -v^2[v(1 - u_4) + uv_4] \tag{s20}$$

$$p = \frac{u}{v(1-u_4)} \tag{s21}$$

(iii) Mapping L^{-1}

$$v_4 = r_4 sin(v_4) + \frac{s_4}{\sqrt{\langle s,s \rangle}} cos(v_4) \tag{s22}$$

$$L = \begin{pmatrix} cos(v_4) & -v^{-1} sin(v_4) \\ sin(v_4) & v^{-1} cos(v_4) \end{pmatrix} \tag{s23}$$

$$u = rcos(v_4) - v^{-1} ssin(v_4) \tag{s24}$$

$$u_4 = r_4 cos(v_4) - v^{-1} s_4 sin(v_4) \tag{s25}$$

$$v = rsin(v_4) + v^{-1} scos(v_4) \tag{s26}$$

$$v_4 = r_4 sin(v_4) + v^{-1} s_4 cos(v_4) \tag{s27}$$

Proposition S2.
(i) $\Phi_{LS}{}^{-1} = S^{-1} \circ L^{-1}$.
(ii) S^{-1} is the inverse of S.
(iii) L^{-1} is the inverse of L.
(iv) $\Phi_{LS}{}^{-1}$ is the inverse of Φ_{LS}.

Proof.
(i) Derivation of $\Phi_{LS}{}^{-1} = S^{-1} \circ L^{-1}$ by substitution.
This is the Cushman and Bates[1] notation for the derivation of $G = G_1 \circ G_2$ in the main paper.
Note: This is exercise 12 in Cushman and Bates[1], which has one typographical error with $<r,r>$ instead of $<s,s>$.

$$q = -v^2[v(1 - u_4) + uv_4]$$

$$\begin{aligned} q = -v^2\big[&rsin(v_4) + v^{-1} scos(v_4)\big(1 - r_4 cos(v_4) + v^{-1} s_4 sin(v_4)\big) \\ &+ \big(rcos(v_4) - v^{-1} ssin(v_4)\big)\big(r_4 sin(v_4) + v^{-1} s_4 cos(v_4)\big)\big] \end{aligned}$$

$$\begin{aligned} q = -v^2[&rsin(v_4) + v^{-1} scos(v_4) - rr_4 sin(v_4) cos(v_4) \\ &- v^{-1} r_4 scos^2(v_4) + v^{-1} rs_4 sin^2(v_4) + v^{-2} ss_4 sin(v_4) cos(v_4) \\ &+ rr_4 sin(v_4) cos(v_4) - v^{-1} r_4 ssin^2(v_4) + v^{-1} rs_4 cos^2(v_4) - v^{-2} ss_4 sin(v_4) cos(v_4)] \end{aligned}$$

$$q = -v^2[rsin(v_4) + v^{-1} scos(v_4) - v^{-1} r_4 s + v^{-1} rs_4]$$

$$q = v^2\big[-r\big(v^{-1} s_4 + sin(v_4)\big) + v^{-1} s\big(r_4 - cos(v_4)\big)\big]$$

$$q = \langle s,s \rangle \left[-r\left(\frac{s_4}{\sqrt{\langle s,s \rangle}} + sin(v_4)\right) + \frac{s}{\sqrt{\langle s,s \rangle}}\big(r_4 - cos(v_4)\big)\right]$$

$$p = \frac{u}{v(1 - v_4)}$$

$$p = \frac{rcos(v_4) - v^{-1} ssin(v_4)}{v\big(1 - r_4 cos(v_4) + v^{-1} s_4 sin(v_4)\big)}$$

$$p = \frac{rcos(v_4) - \frac{s}{\sqrt{\langle s,s\rangle}} sin(v_4)}{\sqrt{\langle s,s\rangle}\left(1 - r_4 cos(v_4) + \frac{s_4}{\sqrt{\langle s,s\rangle}} sin(v_4)\right)}$$

(ii)-(iv) These steps were done in the main paper for the other notation, and a repetition here would not show anything new. ■

Identities

Proof of Proposition 3.
We'll begin with equation (30).

$$r^2 = \sum_{i=0}^{3} r_i^2 = r_0^2 + \boldsymbol{r}\cdot\boldsymbol{r}$$

Then we substitute from mapping F_1, equations (7) and (8)

$$r^2 = (p^2 q - 1)^2 + \left(\sqrt{-2H} q \boldsymbol{p}\right)^2$$

Expand

$$r^2 = q^2 p^4 - 2qp^2 + 1 - 2Hq^2 p^2$$

Substitute the definition of H, equation (1)

$$r^2 = q^2 p^4 - 2qp^2 + 1 - 2\left(\frac{p^2}{2} - \frac{1}{q}\right) q^2 p^2$$

Expand

$$r^2 = q^2 p^4 - 2qp^2 + 1 - q^2 p^4 + 2qp^2$$

Collect similar terms

$$r^2 = 1$$

Then we continue with equation (31)

$$\sum_{i=0}^{3} r_i s_i = r_0 s_0 + \boldsymbol{r}\cdot\boldsymbol{s}$$

Substitute from mapping F_1, equations (7) through (10)

$$\sum_{i=0}^{3} r_i s_i = (p^2 q - 1)\left(-\sqrt{-2H}\boldsymbol{q}\cdot\boldsymbol{p}\right) + \left(\sqrt{-2H} q \boldsymbol{p}\right)\cdot\left(-\left[\frac{\boldsymbol{q}}{q} - (\boldsymbol{q}\cdot\boldsymbol{p})\boldsymbol{p}\right]\right)$$

Expand

$$\sum_{i=0}^{3} r_i s_i = -\sqrt{-2H} q p^2 \boldsymbol{q}\cdot\boldsymbol{p} + \sqrt{-2H}\, \boldsymbol{q}\cdot\boldsymbol{p} - \sqrt{-2H}\boldsymbol{q}\cdot\boldsymbol{p} + \sqrt{-2H} q p^2 \boldsymbol{q}\cdot\boldsymbol{p}$$

Collect similar terms

$$\sum_{i=0}^{3} r_i s_i = 0$$

Then we continue with equation (32)

$$s^2 = \sum_{i=0}^{3} s_i^2 = s_0^2 + \boldsymbol{s} \cdot \boldsymbol{s}$$

Substitute from mapping *F₁*, equations (7) and (10)

$$s^2 = \left(-\sqrt{-2H}\boldsymbol{q}\cdot\boldsymbol{p}\right)^2 + \left(-\left[\frac{\boldsymbol{q}}{q} - (\boldsymbol{q}\cdot\boldsymbol{p})\boldsymbol{p}\right]\right)\cdot\left(-\left[\frac{\boldsymbol{q}}{q} - (\boldsymbol{q}\cdot\boldsymbol{p})\boldsymbol{p}\right]\right)$$

Expand

$$s^2 = -2H(\boldsymbol{q}\cdot\boldsymbol{p})^2 + 1 - \frac{2}{q}(\boldsymbol{q}\cdot\boldsymbol{p})^2 + p^2(\boldsymbol{q}\cdot\boldsymbol{p})^2$$

Substitute the definition of H, equation (1)

$$s^2 = -2\left(\frac{p^2}{2} - \frac{1}{q}\right)(\boldsymbol{q}\cdot\boldsymbol{p})^2 + 1 - \frac{2}{q}(\boldsymbol{q}\cdot\boldsymbol{p})^2 + p^2(\boldsymbol{q}\cdot\boldsymbol{p})^2$$

Expand

$$s^2 = -p^2(\boldsymbol{q}\cdot\boldsymbol{p})^2 + \frac{2}{q}(\boldsymbol{q}\cdot\boldsymbol{p})^2 + 1 - \frac{2}{q}(\boldsymbol{q}\cdot\boldsymbol{p})^2 + p^2(\boldsymbol{q}\cdot\boldsymbol{p})^2$$

Collect similar terms

$$s^2 = 1$$

In contrast with the original paper/thesis, we will confirm the following three identities by using mapping F_2 and the previous three identities. This is much simpler than using the full mapping *F*.

We'll begin with equation (33).

$$\xi^2 = \sum_{i=0}^{3} \xi_i^2 = \xi_0^2 + \boldsymbol{\xi}\cdot\boldsymbol{\xi}$$

Substitute from mapping F_2, equations (13) and (14)

$$\xi^2 = [r_0 cos(\varphi) - s_0\, sin(\varphi)]^2 + [\boldsymbol{r} cos(\varphi) - \boldsymbol{s}\, sin(\varphi)]\cdot[\boldsymbol{r} cos(\varphi) - \boldsymbol{s}\, sin(\varphi)]$$

Expand and collect similar terms

$$\xi^2 = (r_0^2 + \boldsymbol{r}\cdot\boldsymbol{r})cos^2(\varphi) - 2(r_0 s_0 + \boldsymbol{r}\cdot\boldsymbol{s})cos(\varphi)\, sin(\varphi) + (s_0^2 + \boldsymbol{s}\cdot\boldsymbol{s})\, sin^2(\varphi)$$

Use previous identities

$$\xi^2 = cos^2(\varphi) + sin^2(\varphi)$$

Use trigonometric identity

$$\xi^2 = 1$$

Then we continue with equation (34)

$$\sum_{i=0}^{3} \xi_i \eta_i = \xi_0 \eta_0 + \boldsymbol{\xi}\cdot\boldsymbol{\eta}$$

Substitute from mapping F_2, equations (13) through (16)

$$\sum_{i=0}^{3} \xi_i \eta_i = [r_0 cos(\varphi) - s_0\, sin(\varphi)]\left[\frac{1}{\sqrt{-2H}} s_0\, cos(\varphi) + \frac{1}{\sqrt{-2H}} r_0 sin(\varphi)\right] + [\boldsymbol{r} cos(\varphi) - \boldsymbol{s}\, sin(\varphi)]$$
$$\cdot\left[\frac{1}{\sqrt{-2H}}\boldsymbol{s}\, cos(\varphi) + \frac{1}{\sqrt{-2H}}\boldsymbol{r} sin(\varphi)\right]$$

Expand and collect similar terms

$$\sum_{i=0}^{3}\xi_i\eta_i = (r_0 s_0 + \boldsymbol{r}\cdot\boldsymbol{s})\frac{1}{\sqrt{-2H}}cos^2(\varphi) + (r_0^2 + \boldsymbol{r}\cdot\boldsymbol{r})\frac{1}{\sqrt{-2H}}cos(\varphi)\,sin(\varphi) - (s_0^2 + \boldsymbol{s}\cdot\boldsymbol{s})\frac{1}{\sqrt{-2H}}cos(\varphi)\,sin(\varphi) - (r_0 s_0 + \boldsymbol{r}\cdot\boldsymbol{s})\frac{1}{\sqrt{-2H}}sin^2(\varphi)$$

Use previous identities and trigonometric identity

$$\sum_{i=0}^{3}\xi_i\eta_i = 0$$

Then we continue with equation (35)

$$\eta^2 = \sum_{i=0}^{3}\eta_i^2 = \eta_0^2 + \boldsymbol{\eta}\cdot\boldsymbol{\eta}$$

Substitute from mapping F_2, equations (15) and (16)

$$\eta^2 = \left[\frac{1}{\sqrt{-2H}}s_0\,cos(\varphi) + \frac{1}{\sqrt{-2H}}r_0 sin(\varphi)\right]^2 + \left[\frac{1}{\sqrt{-2H}}\boldsymbol{s}\,cos(\varphi) + \frac{1}{\sqrt{-2H}}\boldsymbol{r}sin(\varphi)\right]\cdot\left[\frac{1}{\sqrt{-2H}}\boldsymbol{s}\,cos(\varphi) + \frac{1}{\sqrt{-2H}}\boldsymbol{r}sin(\varphi)\right]$$

Expand and collect similar terms

$$\eta^2 = (s_0^2 + \boldsymbol{s}\cdot\boldsymbol{s})\left(\frac{1}{\sqrt{-2H}}\right)^2 cos^2(\varphi) + 2(r_0 s_0 + \boldsymbol{r}\cdot\boldsymbol{s})\left(\frac{1}{\sqrt{-2H}}\right)^2 cos(\varphi)\,sin(\varphi) + (r_0^2 + \boldsymbol{r}\cdot\boldsymbol{r})\left(\frac{1}{\sqrt{-2H}}\right)^2 sin^2(\varphi)$$

Use previous identities

$$\eta^2 = \left(\frac{1}{-2H}\right)cos^2(\varphi) + \left(\frac{1}{-2H}\right)sin^2(\varphi)$$

Use trigonometric identity

$$\eta^2 = -\frac{1}{2H}$$

Now let's continue with the very important equation (36)

$$\varphi = \sqrt{-2H}\boldsymbol{q}\cdot\boldsymbol{p}$$

Substitute from mapping F_1, equation (11)

$$\varphi = -s_0$$

Now we begin with the right-hand side of equation (36)

$$\varphi = \xi_0 sin(\varphi) - \frac{\eta_0}{\eta}cos(\varphi)$$

Substitute from mapping F_2, equations (13) and (15)

$$\varphi = [r_0 cos(\varphi) - s_0\,sin(\varphi)]sin(\varphi) - \frac{\left[\frac{1}{\sqrt{-2H}}s_0\,cos(\varphi) + \frac{1}{\sqrt{-2H}}r_0 sin(\varphi)\right]}{\eta}cos(\varphi)$$

Use the previous identity of $\eta^2 = -\frac{1}{2H}$

$$\varphi = [r_0 cos(\varphi) - s_0\, sin(\varphi)]sin(\varphi) - s_0\, cos(\varphi) + r_0 sin(\varphi)cos(\varphi)$$

Collect similar terms

$$\varphi = r_0 cos(\varphi)sin(\varphi) - s_0\, sin^2(\varphi) - [s_0 cos^2(\varphi) - r_0 cos(\varphi)]sin(\varphi)$$

Use trigonometric identity

$$\varphi = -s_0$$

Since this identity is so important, we will also calculate it using the full forward mapping. We begin with the right-hand side of equation (36)

$$\varphi = \xi_0 sin(\varphi) - \frac{\eta_0}{\eta} cos(\varphi)$$

Substitute from mapping *F*, equations (3) and (5)

$$\varphi = \left[(p^2 q - 1)cos(\varphi) + \sqrt{-2H}\boldsymbol{q}\cdot\boldsymbol{p}\, sin(\varphi)\right]sin(\varphi) - \frac{\left[-\boldsymbol{q}\cdot\boldsymbol{p}\, cos(\varphi) + \frac{1}{\sqrt{-2H}}(p^2 q - 1)sin(\varphi)\right]}{\eta} cos(\varphi)$$

Use the previous identity of $\eta^2 = -\frac{1}{2H}$

$$\varphi = \left[(p^2 q - 1)cos(\varphi) + \sqrt{-2H}\boldsymbol{q}\cdot\boldsymbol{p}\, sin(\varphi)\right]sin(\varphi) - \left[-\sqrt{-2H}\boldsymbol{q}\cdot\boldsymbol{p}\, cos(\varphi) + (p^2 q - 1)sin(\varphi)\right]cos(\varphi)$$

Expand and collect similar terms

$$\varphi = (p^2 q - 1)cos(\varphi)sin(\varphi) + \sqrt{-2H}\boldsymbol{q}\cdot\boldsymbol{p}\, sin^2(\varphi) + \sqrt{-2H}\boldsymbol{q}\cdot\boldsymbol{p}\, cos^2(\varphi) - (p^2 q - 1)\, cos(\varphi)sin(\varphi)$$

Use trigonometric identity

$$\varphi = \sqrt{-2H}\boldsymbol{q}\cdot\boldsymbol{p}$$

The next identity, equation (37), is important, because it appears as a denominator in equation (29) of the inverse LS mapping.

$$-2Hq = -p^2 q + 2 = -(\boldsymbol{p}\cdot\boldsymbol{p})\sqrt{\boldsymbol{q}\cdot\boldsymbol{q}} + 2$$

Substitute from mapping G_1, equations (24) and (26)

$$-2Hq = -\left(\frac{\sqrt{-2H}\boldsymbol{r}}{(1-r_0)}\right)\cdot\left(\frac{\sqrt{-2H}\boldsymbol{r}}{(1-r_0)}\right)\sqrt{\left(\frac{1}{2H}[\boldsymbol{s}(1-r_0)+\boldsymbol{r}s_0]\right)\cdot\left(\frac{1}{2H}[\boldsymbol{s}(1-r_0)+\boldsymbol{r}s_0]\right)} + 2$$

Expand

$$-2Hq = \frac{\boldsymbol{r}\cdot\boldsymbol{r}\sqrt{\boldsymbol{s}\cdot\boldsymbol{s}(1-r_0)^2 + 2\boldsymbol{r}\cdot\boldsymbol{s}(1-r_0)s_0 + \boldsymbol{r}\cdot\boldsymbol{r}{s_0}^2}}{(1-r_0)^2} + 2$$

Use previous identities

$$-2Hq = \frac{(1-{r_0}^2)\sqrt{(1-{s_0}^2)(1-r_0)^2 + 2(-r_0 s_0)(1-r_0)s_0 + (1-{r_0}^2){s_0}^2}}{(1-r_0)^2} + 2$$

Expand

$$-2Hq = \frac{(1-{r_0}^2)}{(1-r_0)^2}\sqrt{1 - 2r_0 + {r_0}^2 - {s_0}^2 + 2r_0{s_0}^2 - {r_0}^2{s_0}^2 - 2r_0{s_0}^2 + 2{r_0}^2{s_0}^2 + {s_0}^2 - {r_0}^2{s_0}^2} + 2$$

Collect similar terms

$$-2Hq = \frac{(1 - r_0{}^2)}{(1 - r_0)^2}\sqrt{1 - 2r_0 + r_0{}^2} + 2$$

Resolve square root, choosing a minus sign.

$$-2Hq = \frac{-(1 - r_0{}^2)}{(1 - r_0)} + 2$$

Expand

$$-2Hq = \frac{-1 + r_0{}^2 + 2 - 2r_0}{1 - r_0} = \frac{1 - 2r_0 + r_0{}^2}{1 - r_0} = 1 - r_0$$

Substitute from mapping *G₂*, equation (19)

$$-2Hq = 1 - r_0 = 1 - \xi_0 cos(\varphi) - \frac{\eta_0}{\eta} sin(\varphi)$$

Then, we continue with the identity for angular momentum (38).

$$L_i = q_j p_k - q_k p_j$$

Substitute from mapping G_1, equations (24) and (26)

$$L_i = \left(\frac{1}{2H}\left[s_j(1 - r_0) + r_j s_0\right]\right)\left(\frac{\sqrt{-2H} r_k}{(1 - r_0)}\right) - \left(\frac{1}{2H}\left[s_k(1 - r_0) + r_k s_0\right]\right)\left(\frac{\sqrt{-2H} r_j}{(1 - r_0)}\right)$$

Expand

$$L_i = \frac{1}{\sqrt{-2H}(1 - r_0)}\left[-r_k s_j(1 - r_0) - r_j r_k s_0 + r_j s_k(1 - r_0) + r_j r_k s_0\right]$$

Collect similar terms

$$L_i = \frac{1}{\sqrt{-2H}}\left(r_j s_k - r_k s_j\right)$$

Substitute from mapping G_2, equations (20) and (22)

$$L_i = \frac{1}{\sqrt{-2H}}\left[\left(\xi_j cos(\varphi) + \frac{\eta_j}{\eta} sin(\varphi)\right)\left(-\xi_k sin(\varphi) + \frac{\eta_k}{\eta} cos(\varphi)\right) - \left(\xi_k cos(\varphi) + \frac{\eta_k}{\eta} sin(\varphi)\right)\left(-\xi_j sin(\varphi) + \frac{\eta_j}{\eta} cos(\varphi)\right)\right]$$

Expand

$$L_i = \frac{1}{\sqrt{-2H}}\left(-\xi_j \xi_k cos(\varphi) sin(\varphi) + \frac{\xi_j \eta_k}{\eta} cos^2(\varphi) - \frac{\xi_k \eta_j}{\eta} sin^2(\varphi) + \frac{\eta_j \eta_k}{\eta^2} cos(\varphi) sin(\varphi) + \xi_j \xi_k cos(\varphi) sin(\varphi) - \frac{\xi_k \eta_j}{\eta} cos^2(\varphi) + \frac{\xi_j \eta_k}{\eta} sin^2(\varphi) - \frac{\eta_j \eta_k}{\eta^2} cos(\varphi) sin(\varphi)\right)$$

Collect similar terms

$$L_i = \frac{1}{\sqrt{-2H}}\left(\frac{\xi_j \eta_k}{\eta} - \frac{\xi_k \eta_j}{\eta}\right)\left(cos^2(\varphi) + sin^2(\varphi)\right)$$

Use the previous identity of $\eta^2 = -\frac{1}{2H}$ and trigonometric identity

$$L_i = \left(\xi_j \eta_k - \xi_k \eta_j\right)$$

Continue with the identity for the rescaled Runge-Lenz vector (39).

$$M_i = \frac{1}{\sqrt{-2H}}\left(\frac{q_i}{q} + p_i(\boldsymbol{q}\cdot\boldsymbol{p}) - q_i p^2\right)$$

Use previous identity for $\varphi = \sqrt{-2H}\boldsymbol{q}\cdot\boldsymbol{p} = -s_0$

$$M_i = \frac{1}{\sqrt{-2H}}\left(\frac{q_i}{q} + p_i\left(\frac{-s_0}{\sqrt{-2H}}\right) - q_i p^2\right)$$

Substitute interim result for q from equation (23), i.e. equation (7)

$$M_i = \frac{1}{\sqrt{-2H}}\left(\frac{-2H}{(1-r_0)}q_i + p_i\left(\frac{-s_0}{\sqrt{-2H}}\right) - q_i p^2\right)$$

Substitute from mapping *G₁*, equations (24) and (26)

$$M_i = \frac{1}{\sqrt{-2H}}\left[\left(\frac{-2H}{1-r_0}\right)\left(\frac{1}{2H}[s_i(1-r_0)+r_i s_0]\right) + \left(\frac{-s_0}{\sqrt{-2H}}\right)\left(\frac{\sqrt{-2H}r_i}{1-r_0}\right) - \left(\frac{1}{2H}[s_i(1-r_0)+r_i s_0]\right)\left(\frac{\sqrt{-2H}\boldsymbol{r}}{1-r_0}\right)\cdot\left(\frac{\sqrt{-2H}\boldsymbol{r}}{1-r_0}\right)\right]$$

Collect similar terms

$$M_i = \frac{1}{\sqrt{-2H}}\left[-s_i - \frac{2r_i s_0}{1-r_0} + [s_i(1-r_0)+r_i s_0]\frac{(\boldsymbol{r}\cdot\boldsymbol{r})}{(1-r_0)^2}\right]$$

Use previous identity

$$M_i = \frac{1}{\sqrt{-2H}}\left[-s_i - \frac{2r_i s_0}{1-r_0} + [s_i(1-r_0)+r_i s_0]\frac{(1-{r_0}^2)}{(1-r_0)^2}\right]$$

Expand

$$M_i = \frac{1}{\sqrt{-2H}}\frac{1}{(1-r_0)^2}[-s_i(1-r_0)^2 - 2r_i s_0(1-r_0) + [s_i(1-r_0)+r_i s_0](1-{r_0}^2)]$$

Expand

$$M_i = \frac{1}{\sqrt{-2H}}\frac{1}{(1-r_0)^2}[-s_i + 2s_i r_0 - s_i {r_0}^2 - 2r_i s_0 + 2r_0 r_i s_0 + s_i - {r_0}^2 s_i - r_0 s_i + {r_0}^3 s_i + r_i s_0 - {r_0}^2 r_i s_0]$$

Collect similar terms

$$M_i = \frac{1}{\sqrt{-2H}}\frac{1}{(1-r_0)^2}[r_0 s_i - 2{r_0}^2 s_i - r_i s_0 + 2r_0 r_i s_0 - {r_0}^2 r_i s_0 + {r_0}^3 s_i]$$

$$M_i = \frac{1}{\sqrt{-2H}}\frac{1}{(1-r_0)^2}[r_0 s_i(1-2r_0+{r_0}^2) - r_i s_0(1-2r_0+{r_0}^2)]$$

$$M_i = \frac{1}{\sqrt{-2H}}(r_0 s_i - r_i s_0)$$

Substitute from mapping *G₂*, equations (19) through (22)

$$M_i = \frac{1}{\sqrt{-2H}}\left[\left(\xi_0 cos(\varphi) + \frac{\eta_0}{\eta}sin(\varphi)\right)\left(-\xi_i sin(\varphi) + \frac{\eta_k}{\eta}cos(\varphi)\right) - \left(\xi_i cos(\varphi) + \frac{\eta_i}{\eta}sin(\varphi)\right)\left(-\xi_0 sin(\varphi) + \frac{\eta_0}{\eta}cos(\varphi)\right)\right]$$

Expand

$$M_i = \frac{1}{\sqrt{-2H}}\left(-\xi_0\xi_i cos(\varphi)sin(\varphi) + \frac{\xi_0\eta_i}{\eta}cos^2(\varphi) - \frac{\xi_i\eta_0}{\eta}sin^2(\varphi) + \frac{\eta_0\eta_i}{\eta^2}cos(\varphi)sin(\varphi) + \xi_0\xi_i cos(\varphi)sin(\varphi) - \frac{\xi_i\eta_0}{\eta}cos^2(\varphi) + \frac{\xi_0\eta_i}{\eta}sin^2(\varphi) - \frac{\eta_0\eta_i}{\eta^2}cos(\varphi)sin(\varphi)\right)$$

Collect similar terms

$$M_i = \frac{1}{\sqrt{-2H}}\left(\frac{\xi_0\eta_i}{\eta} - \frac{\xi_i\eta_0}{\eta}\right)\left(cos^2(\varphi) + sin^2(\varphi)\right)$$

Use the previous identity of $\eta^2 = -\frac{1}{2H}$ and trigonometric identity

$$M_i = (\xi_0\eta_i - \xi_i\eta_0)$$

The next identity, equation (40), is just a restatement of the previous identity for η^2.

Continue with the Hamiltonian of the rotating Kepler problem (41). This is just a combination of the previous identities for the Kepler Hamiltonian and the angular momentum and requires no further calculation. This completes the proof of Proposition 3. ■

Positive Energy

Proof of Proposition 5.

(i) Derivation of $G = G_1 \circ G_2$ by substitution.

We begin with the definition of $\boldsymbol{q}$ from G_1,

$$\boldsymbol{q} = -\frac{1}{2H}[\boldsymbol{s}(1 - r_0) + \boldsymbol{r}s_0]$$

substitute the values or $\boldsymbol{r}$ and $\boldsymbol{s}$ from G_2,

$$\boldsymbol{q} = \frac{1}{2H}\left[\left(-\boldsymbol{\xi} sinh(\varphi) - \frac{\boldsymbol{\eta}}{\sqrt{-\eta^2}}cosh(\varphi)\right)\left(1 - \xi_0 cosh(\varphi) - \frac{\eta_0}{\sqrt{-\eta^2}}sinh(\varphi)\right) + \left(\boldsymbol{\xi} cosh(\varphi) + \frac{\boldsymbol{\eta}}{\sqrt{-\eta^2}}sinh(\varphi)\right)\left(-\xi_0 sinh(\varphi) - \frac{\eta_0}{\sqrt{-\eta^2}}cosh(\varphi)\right)\right]$$

expand the expression,

$$\boldsymbol{q} = \frac{1}{2H}\left[-\boldsymbol{\xi} sinh(\varphi) - \frac{\boldsymbol{\eta}}{\sqrt{-\eta^2}}cosh(\varphi) + \boldsymbol{\xi}\xi_0 sinh(\varphi)cosh(\varphi) + \xi_0\frac{\boldsymbol{\eta}}{\sqrt{-\eta^2}}cosh^2(\varphi) + \boldsymbol{\xi}\frac{\eta_0}{\sqrt{-\eta^2}}sinh^2(\varphi) - \frac{\boldsymbol{\eta}\eta_0}{\eta^2}sinh(\varphi)cosh(\varphi) - \boldsymbol{\xi}\xi_0 sinh(\varphi)cosh(\varphi) - \xi_0\frac{\boldsymbol{\eta}}{\sqrt{-\eta^2}}sinh^2(\varphi) - \boldsymbol{\xi}\frac{\eta_0}{\sqrt{-\eta^2}}cosh^2(\varphi) + \frac{\boldsymbol{\eta}\eta_0}{\eta^2}sinh(\varphi)cosh(\varphi)\right]$$

collect similar terms and use a trigonometric identity,

$$\boldsymbol{q} = \frac{1}{2H}\left[-\boldsymbol{\xi} sinh(\varphi) - \frac{\boldsymbol{\eta}}{\sqrt{-\eta^2}}cosh(\varphi) + \xi_0\frac{\boldsymbol{\eta}}{\sqrt{-\eta^2}} - \boldsymbol{\xi}\frac{\eta_0}{\sqrt{-\eta^2}}\right]$$

use the previous identity of $\eta^2 = -\frac{1}{2H}$

$$\boldsymbol{q} = -\eta^2\left[-\boldsymbol{\xi}\left(\frac{\eta_0}{\sqrt{-\eta^2}} + sinh(\varphi)\right) + \frac{\boldsymbol{\eta}}{\sqrt{-\eta^2}}\left(\xi_0 - cosh(\varphi)\right)\right]$$

Now we take the definition of $\boldsymbol{p}$ from G_1.

$$\boldsymbol{p} = -\frac{\sqrt{2H}\boldsymbol{r}}{(1-r_0)}$$

and substitute the values or $\boldsymbol{r}$ and $\boldsymbol{s}$ from G_2.

$$\boldsymbol{p} = -\frac{\left(\boldsymbol{\xi} cosh(\varphi) + \frac{\boldsymbol{\eta}}{\sqrt{-\eta^2}} sinh(\varphi)\right)}{\sqrt{-\eta^2}\left(1 - \xi_0 cosh(\varphi) - \frac{\eta_0}{\sqrt{-\eta^2}} sinh(\varphi)\right)}$$

(ii) Derivation of $\boldsymbol{G_1} = \boldsymbol{F_1^{-1}}$

We begin with F_1:

$$r_0 = p^2 q - 1 \tag{47}$$

$$\boldsymbol{r} = \sqrt{2H} q \boldsymbol{p} \tag{48}$$

$$s_0 = -\sqrt{2H} \boldsymbol{q} \cdot \boldsymbol{p} \tag{49}$$

$$\boldsymbol{s} = \left[\frac{\boldsymbol{q}}{q} - (\boldsymbol{q} \cdot \boldsymbol{p})\boldsymbol{p}\right] \tag{50}$$

Now we can solve (47) for q:

$$r_0 - 1 = p^2 q - 2 = 2\mathrm{q}\left(\frac{p^2}{2} - \frac{1}{q}\right) = 2Hq.$$

$$q = \frac{1}{-2H}(1 - r_0)$$

Then we can solve (48) for $\boldsymbol{p}$:

$$\boldsymbol{p} = \frac{\boldsymbol{r}}{\sqrt{2H}q} = -\sqrt{2H}\frac{\boldsymbol{r}}{(1-r_0)}.$$

$$\boldsymbol{p} = -\sqrt{2H}\frac{\boldsymbol{r}}{(1-r_0)}$$

With this, we can solve (50) for $\boldsymbol{q}$:

$$\boldsymbol{q} = q\boldsymbol{s} + (\boldsymbol{q} \cdot \boldsymbol{p})q\boldsymbol{p}$$

substitute q, $\boldsymbol{q} \cdot \boldsymbol{p}$ and $\boldsymbol{p}$ from above and (49),

$$\boldsymbol{q} = -\frac{1}{2H}(1 - r_0)\boldsymbol{s} + \frac{s_0}{-\sqrt{2H}}\left(\frac{1}{-2H}\right)(1 - r_0)\left(-\sqrt{2H}\frac{\boldsymbol{r}}{1 - r_0}\right)$$

and simplify.

$$\boldsymbol{q} = -\frac{1}{2H}[(1 - r_0)\boldsymbol{s} + s_0\boldsymbol{r}]$$

The result is now mapping G_1.

(iii) and (iv) This is analogous to the case of negative energy. This completes the proof of Proposition 5. ■

Identities

Proof of Proposition 6.

We'll begin with equation (69).

$$r^2 = r_0^2 - \sum_{i=1}^{3} r_i^2 = r_0^2 - \boldsymbol{r} \cdot \boldsymbol{r}$$

Then we substitute from mapping F_1, equations (47) and (48)

$$r^2 = (p^2 q - 1)^2 - \left(\sqrt{2H} q \boldsymbol{p}\right)^2$$

Expand

$$r^2 = q^2 p^4 - 2qp^2 + 1 - 2Hq^2 p^2$$

Substitute the definition of H, equation (1)

$$r^2 = q^2 p^4 - 2qp^2 + 1 - 2\left(\frac{p^2}{2} - \frac{1}{q}\right) q^2 p^2$$

Expand

$$r^2 = q^2 p^4 - 2qp^2 + 1 - q^2 p^4 + 2qp^2$$

Collect similar terms

$$r^2 = 1$$

Then we continue with equation (69)

$$r_0 s_0 - \sum_{i=1}^{3} r_i s_i = r_0 s_0 - \boldsymbol{r} \cdot \boldsymbol{s}$$

Substitute from mapping F_1, equations (47) through (50)

$$r_0 s_0 - \sum_{i=1}^{3} r_i s_i = (p^2 q - 1)\left(-\sqrt{2H} \boldsymbol{q} \cdot \boldsymbol{p}\right) - \left(\sqrt{2H} q \boldsymbol{p}\right) \cdot \left(\left[\frac{\boldsymbol{q}}{q} - (\boldsymbol{q} \cdot \boldsymbol{p})\boldsymbol{p}\right]\right)$$

Expand

$$r_0 s_0 - \sum_{i=1}^{3} r_i s_i = -\sqrt{2H} q p^2 \boldsymbol{q} \cdot \boldsymbol{p} + \sqrt{2H}\, \boldsymbol{q} \cdot \boldsymbol{p} - \sqrt{2H} \boldsymbol{q} \cdot \boldsymbol{p} + \sqrt{2H} q p^2 \boldsymbol{q} \cdot \boldsymbol{p}$$

Collect similar terms

$$r_0 s_0 - \sum_{i=1}^{3} r_i s_i = 0$$

Then we continue with equation (70)

$$s^2 = s_0^2 - \sum_{i=1}^{3} s_i^2 = s_0^2 - \boldsymbol{s} \cdot \boldsymbol{s}$$

Substitute from mapping F_1, equations (49) and (50)

$$s^2 = \left(-\sqrt{2H} \boldsymbol{q} \cdot \boldsymbol{p}\right)^2 - \left(\left[\frac{\boldsymbol{q}}{q} - (\boldsymbol{q} \cdot \boldsymbol{p})\boldsymbol{p}\right]\right) \cdot \left(\left[\frac{\boldsymbol{q}}{q} - (\boldsymbol{q} \cdot \boldsymbol{p})\boldsymbol{p}\right]\right)$$

Expand

$$s^2 = 2H(\boldsymbol{q} \cdot \boldsymbol{p})^{\mathbf{2}} - 1 + \frac{2}{q}(\boldsymbol{q} \cdot \boldsymbol{p})^{\mathbf{2}} - p^2 (\boldsymbol{q} \cdot \boldsymbol{p})^2$$

Substitute the definition of H, equation (1)

$$s^2 = 2\left(\frac{p^2}{2} - \frac{1}{q}\right)(\boldsymbol{q}\cdot\boldsymbol{p})^2 - 1 + \frac{2}{q}(\boldsymbol{q}\cdot\boldsymbol{p})^2 - p^2(\boldsymbol{q}\cdot\boldsymbol{p})^2$$

Expand

$$s^2 = p^2(\boldsymbol{q}\cdot\boldsymbol{p})^2 - \frac{2}{q}(\boldsymbol{q}\cdot\boldsymbol{p})^2 - 1 + \frac{2}{q}(\boldsymbol{q}\cdot\boldsymbol{p})^2 - p^2(\boldsymbol{q}\cdot\boldsymbol{p})^2$$

Collect similar terms

$$s^2 = -1$$

In contrast with the original paper/thesis, we will confirm the following three identities by using mapping F_2 and the previous three identities. This is much simpler than using the full mapping F.

We'll begin with equation (71).

$$\xi^2 = \xi_0^2 - \sum_{i=1}^{3} \xi_i^2 = \xi_0^2 - \boldsymbol{\xi}\cdot\boldsymbol{\xi}$$

Substitute from mapping F_2, equations (53) and (54)

$$\xi^2 = [r_0 cosh(\varphi) + s_0\, sinh(\varphi)]^2 - [\boldsymbol{r} cosh(\varphi) + \boldsymbol{s}\, sinh(\varphi)]\cdot[\boldsymbol{r} cosh(\varphi) + \boldsymbol{s}\, sinh(\varphi)]$$

Expand and collect similar terms

$$\xi^2 = (r_0^2 - \boldsymbol{r}\cdot\boldsymbol{r}) cosh^2(\varphi) + 2(r_0 s_0 - \boldsymbol{r}\cdot\boldsymbol{s}) cosh(\varphi)\, sinh(\varphi) + (s_0^2 - \boldsymbol{s}\cdot\boldsymbol{s})\, sinh^2(\varphi)$$

Use previous identities

$$\xi^2 = cosh^2(\varphi) - sinh^2(\varphi)$$

Use trigonometric identity

$$\xi^2 = 1$$

Then we continue with equation (72)

$$\xi_0\eta_0 - \sum_{i=1}^{3} \xi_i\eta_i = \xi_0\eta_0 - \boldsymbol{\xi}\cdot\boldsymbol{\eta}$$

Substitute from mapping F_2, equations (53) through (56)

$$\xi_0\eta_0 - \sum_{i=1}^{3} \xi_i\eta_i = [r_0 cosh(\varphi) + s_0\, sinh(\varphi)]\left[-\frac{1}{\sqrt{2H}} s_0\, cosh(\varphi) - \frac{1}{\sqrt{2H}} r_0 sinh(\varphi)\right] - [\boldsymbol{r} cosh(\varphi) + \boldsymbol{s}\, sinh(\varphi)]\cdot\left[-\frac{1}{\sqrt{2H}}\boldsymbol{s}\, cosh(\varphi) - \frac{1}{\sqrt{2H}}\boldsymbol{r} sinh(\varphi)\right]$$

Expand and collect similar terms

$$\xi_0\eta_0 - \sum_{i=1}^{3} \xi_i\eta_i = -(r_0 s_0 - \boldsymbol{r}\cdot\boldsymbol{s})\frac{1}{\sqrt{2H}} cosh^2(\varphi) - (r_0^2 - \boldsymbol{r}\cdot\boldsymbol{r})\frac{1}{\sqrt{2H}} cosh(\varphi)\, sinh(\varphi) - (s_0^2 - \boldsymbol{s}\cdot\boldsymbol{s})\frac{1}{\sqrt{2H}} cosh(\varphi)\, sinh(\varphi) - (r_0 s_0 - \boldsymbol{r}\cdot\boldsymbol{s})\frac{1}{\sqrt{2H}} sinh^2(\varphi)$$

Use previous identities

$$\xi_0\eta_0 - \sum_{i=1}^{3} \xi_i\eta_i = 0$$

Then we continue with equation (73)

$$\eta^2 = \eta_0^2 - \sum_{i=1}^{3} \eta_i^2 = \eta_0^2 - \boldsymbol{\eta} \cdot \boldsymbol{\eta}$$

Substitute from mapping F_2, equations (55) and (56)

$$\eta^2 = \left[-\frac{1}{\sqrt{2H}} s_0\, cosh(\varphi) - \frac{1}{\sqrt{2H}} r_0 sinh(\varphi)\right]^2 - \left[-\frac{1}{\sqrt{2H}} \boldsymbol{s}\, cosh(\varphi) - \frac{1}{\sqrt{2H}} \boldsymbol{r} sinh(\varphi)\right] \cdot \left[-\frac{1}{\sqrt{2H}} \boldsymbol{s}\, cosh(\varphi) - \frac{1}{\sqrt{2H}} \boldsymbol{r} sinh(\varphi)\right]$$

Expand and collect similar terms

$$\eta^2 = (s_0^2 - \boldsymbol{s} \cdot \boldsymbol{s}) \left(\frac{1}{\sqrt{2H}}\right)^2 cosh^2(\varphi) + 2(r_0 s_0 - \boldsymbol{r} \cdot \boldsymbol{s}) \left(\frac{1}{\sqrt{2H}}\right)^2 cosh(\varphi)\, sinh(\varphi) + (r_0^2 - \boldsymbol{r} \cdot \boldsymbol{r}) \left(\frac{1}{\sqrt{2H}}\right)^2 sinh^2(\varphi)$$

Use previous identities

$$\eta^2 = -\left(\frac{1}{2H}\right) cosh^2(\varphi) + \left(\frac{1}{2H}\right) sinh^2(\varphi)$$

Use trigonometric identity

$$\eta^2 = -\frac{1}{2H}$$

Now let's continue with the very important equation (74)

$$\varphi = \sqrt{2H} \boldsymbol{q} \cdot \boldsymbol{p}$$

Substitute from mapping F_1, equation (49)

$$\varphi = -s_0$$

Now we begin with the right-hand side of equation (74)

$$\varphi = \xi_0 sinh(\varphi) + \frac{\eta_0}{\sqrt{-\eta^2}} cosh(\varphi)$$

Substitute from mapping F_2, equations (43) and (45)

$$\varphi = [r_0 cosh(\varphi) + s_0\, sinh(\varphi)] sinh(\varphi) - \frac{\left[\frac{1}{\sqrt{2H}} s_0\, cosh(\varphi) + \frac{1}{\sqrt{2H}} r_0 sinh(\varphi)\right]}{\sqrt{-\eta^2}} cosh(\varphi)$$

Use the previous identity of $\eta^2 = -\frac{1}{2H}$

$$\varphi = [r_0 cosh(\varphi) + s_0\, sinh(\varphi)] sinh(\varphi) - [s_0\, cosh(\varphi) + r_0 sinh(\varphi)] cosh(\varphi)$$

Expand and collect similar terms

$$\varphi = r_0 cosh(\varphi) sinh(\varphi) + s_0\, sinh^2(\varphi) - s_0 cosh^2(\varphi) - r_0 cosh(\varphi) sinh(\varphi)$$

Use trigonometric identity

$$\varphi = -s_0$$

Since this identity is so important, we will also calculate it using the full forward mapping. We begin with the right-hand side of equation (74)

$$\varphi = \xi_0 sinh(\varphi) + \frac{\eta_0}{\sqrt{-\eta^2}} cosh(\varphi)$$

Substitute from mapping *F*, equations (43) and (45)

$$\varphi = \left[(p^2q-1)cosh(\varphi) - \sqrt{2H}\boldsymbol{q}\cdot\boldsymbol{p}\, sinh(\varphi)\right]sinh(\varphi) + \frac{\left[\boldsymbol{q}\cdot\boldsymbol{p}\, cosh(\varphi) - \frac{1}{\sqrt{2H}}(p^2q-1)sinh(\varphi)\right]}{\sqrt{-\eta^2}} cosh(\varphi)$$

Use the previous identity of $\eta^2 = -\frac{1}{2H}$

$$\varphi = \left[(p^2q-1)cosh(\varphi) - \sqrt{2H}\boldsymbol{q}\cdot\boldsymbol{p}\, sinh(\varphi)\right]sinh(\varphi) + \left[\sqrt{2H}\boldsymbol{q}\cdot\boldsymbol{p}\, cosh(\varphi) - (p^2q-1)sinh(\varphi)\right]cosh(\varphi)$$

Expand and collect similar terms

$$\varphi = (p^2q-1)cosh(\varphi)sinh(\varphi) - \sqrt{2H}\boldsymbol{q}\cdot\boldsymbol{p}\, sinh^2(\varphi) + \sqrt{2H}\boldsymbol{q}\cdot\boldsymbol{p}\, cosh^2(\varphi) - (p^2q-1)\, cosh(\varphi)sinh(\varphi)$$

Use trigonometric identity

$$\varphi = \sqrt{2H}\boldsymbol{q}\cdot\boldsymbol{p}$$

The next identity, equation (75), is important, because it appears as a denominator in equation (67) of the inverse LS mapping.

$$-2Hq = -p^2q + 2 = -(\boldsymbol{p}\cdot\boldsymbol{p})\sqrt{\boldsymbol{q}\cdot\boldsymbol{q}} + 2$$

Substitute from mapping G_1, equations (63) and (64)

$$-2Hq = -\left(-\frac{\sqrt{2H}\boldsymbol{r}}{(1-r_0)}\right)\cdot\left(-\frac{\sqrt{2H}\boldsymbol{r}}{(1-r_0)}\right)\sqrt{\left(-\frac{1}{2H}[\boldsymbol{s}(1-r_0)+\boldsymbol{r}s_0]\right)\cdot\left(-\frac{1}{2H}[\boldsymbol{s}(1-r_0)+\boldsymbol{r}s_0]\right)} + 2$$

Expand

$$-2Hq = -\frac{\boldsymbol{r}\cdot\boldsymbol{r}\sqrt{\boldsymbol{s}\cdot\boldsymbol{s}(1-r_0)^2 + 2\boldsymbol{r}\cdot\boldsymbol{s}(1-r_0)s_0 + \boldsymbol{r}\cdot\boldsymbol{r}{s_0}^2}}{(1-r_0)^2} + 2$$

Use previous identities

$$-2Hq = \frac{(1-{r_0}^2)\sqrt{(1+{s_0}^2)(1-r_0)^2 + 2(r_0s_0)(1-r_0)s_0 - (1-{r_0}^2){s_0}^2}}{(1-r_0)^2} + 2$$

Expand

$$-2Hq = \frac{(1-{r_0}^2)}{(1-r_0)^2}\sqrt{1-2r_0+{r_0}^2+{s_0}^2-2r_0{s_0}^2+{r_0}^2{s_0}^2+2r_0{s_0}^2-2{r_0}^2{s_0}^2-{s_0}^2+{r_0}^2{s_0}^2} + 2$$

Collect similar terms

$$-2Hq = \frac{(1-{r_0}^2)}{(1-r_0)^2}\sqrt{1-2r_0+{r_0}^2} + 2$$

Resolve square root, choosing a minus sign.

$$-2Hq = \frac{-(1-{r_0}^2)}{(1-r_0)} + 2$$

Expand

$$-2Hq = \frac{-1 + {r_0}^2 + 2 - 2r_0}{1 - r_0} = \frac{1 - 2r_0 + {r_0}^2}{1 - r_0} = 1 - r_0$$

Substitute from mapping G_2, equation (59)

$$-2Hq = 1 - r_0 = 1 - \xi_0 cosh(\varphi) - \frac{\eta_0}{\sqrt{-\eta^2}} sinh(\varphi)$$

Then, we continue with the identity for angular momentum (76).

$$L_i = q_j p_k - q_k p_j$$

Substitute from mapping G_1, equations (63) and (64)

$$L_i = \left(-\frac{1}{2H}\left[s_j(1 - r_0) + r_j s_0\right]\right)\left(-\frac{\sqrt{2H} r_k}{(1 - r_0)}\right) - \left(-\frac{1}{2H}\left[s_k(1 - r_0) + r_k s_0\right]\right)\left(-\frac{\sqrt{2H} r_j}{(1 - r_0)}\right)$$

Expand

$$L_i = \frac{1}{\sqrt{2H}(1 - r_0)}\left[r_k s_j(1 - r_0) + r_j r_k s_0 - r_j s_k(1 - r_0) - r_j r_k s_0\right]$$

Collect similar terms

$$L_i = -\frac{1}{\sqrt{2H}}\left(r_j s_k - r_k s_j\right)$$

Substitute from mapping G_2, equations (60) and (62)

$$L_i = -\frac{1}{\sqrt{2H}}\left[\left(\xi_j cosh(\varphi) + \frac{\eta_j}{\sqrt{-\eta^2}} sinh(\varphi)\right)\left(-\xi_k sinh(\varphi) - \frac{\eta_k}{\sqrt{-\eta^2}} cosh(\varphi)\right) - \left(\xi_k cosh(\varphi) + \frac{\eta_k}{\sqrt{-\eta^2}} sinh(\varphi)\right)\left(-\xi_j sinh(\varphi) - \frac{\eta_j}{\sqrt{-\eta^2}} cosh(\varphi)\right)\right]$$

Expand

$$L_i = -\frac{1}{\sqrt{2H}}\left(-\xi_j \xi_k cosh(\varphi) sinh(\varphi) - \frac{\xi_j \eta_k}{\sqrt{-\eta^2}} cosh^2(\varphi) - \frac{\xi_k \eta_j}{\sqrt{-\eta^2}} sinh^2(\varphi) + \frac{\eta_j \eta_k}{\eta^2} cosh(\varphi) sinh(\varphi) + \xi_j \xi_k cosh(\varphi) sinh(\varphi) + \frac{\xi_k \eta_j}{\sqrt{-\eta^2}} cosh^2(\varphi) + \frac{\xi_j \eta_k}{\sqrt{-\eta^2}} sinh^2(\varphi) - \frac{\eta_j \eta_k}{\eta^2} cosh(\varphi) sinh(\varphi)\right)$$

Collect similar terms

$$L_i = \frac{1}{\sqrt{2H}}\left(\frac{\xi_j \eta_k}{\sqrt{-\eta^2}} - \frac{\xi_k \eta_j}{\sqrt{-\eta^2}}\right)\left(cosh^2(\varphi) - sinh^2(\varphi)\right)$$

Use the previous identity of $\eta^2 = -\frac{1}{2H}$ and trigonometric identity

$$L_i = \left(\xi_j \eta_k - \xi_k \eta_j\right)$$

Continue with the identity for the rescaled Runge-Lenz vector (77).

$$M_i = \frac{1}{\sqrt{2H}}\left(\frac{q_i}{q} + p_i(\boldsymbol{q}\cdot\boldsymbol{p}) - q_i p^2\right)$$

Use previous identity for $\varphi = \sqrt{2H}\boldsymbol{q}\cdot\boldsymbol{p} = -s_0$

$$M_i = \frac{1}{\sqrt{2H}}\left(\frac{q_i}{q} + p_i\left(\frac{-s_0}{\sqrt{2H}}\right) - q_i p^2\right)$$

Substitute interim result for q from equation (47)

$$M_i = \frac{1}{\sqrt{2H}}\left(\frac{-2H}{(1-r_0)}q_i + p_i\left(\frac{-s_0}{\sqrt{2H}}\right) - q_i p^2\right)$$

Substitute from mapping *G₁*, equations (63) and (64)

$$M_i = \frac{1}{\sqrt{2H}}\left[\left(\frac{-2H}{1-r_0}\right)\left(-\frac{1}{2H}[s_i(1-r_0)+r_i s_0]\right) + \left(\frac{-s_0}{\sqrt{2H}}\right)\left(-\frac{\sqrt{2H}r_i}{1-r_0}\right) - \left(-\frac{1}{2H}[s_i(1-r_0)+r_i s_0]\right)\left(-\frac{\sqrt{2H}\boldsymbol{r}}{1-r_0}\right)\cdot\left(-\frac{\sqrt{2H}\boldsymbol{r}}{1-r_0}\right)\right]$$

Expand and collect similar terms

$$M_i = \frac{1}{\sqrt{2H}}\left[s_i + \frac{2r_i s_0}{1-r_0} + [s_i(1-r_0)+r_i s_0]\frac{(\boldsymbol{r}\cdot\boldsymbol{r})}{(1-r_0)^2}\right]$$

Use previous identity

$$M_i = \frac{1}{\sqrt{2H}}\left[s_i + \frac{2r_i s_0}{1-r_0} - [s_i(1-r_0)+r_i s_0]\frac{(1-{r_0}^2)}{(1-r_0)^2}\right]$$

Expand

$$M_i = \frac{1}{\sqrt{2H}}\frac{1}{(1-r_0)^2}[s_i(1-r_0)^2 + 2r_i s_0(1-r_0) - [s_i(1-r_0)+r_i s_0](1-{r_0}^2)]$$

Expand

$$M_i = \frac{1}{\sqrt{2H}}\frac{1}{(1-r_0)^2}[s_i - 2s_i r_0 + s_i {r_0}^2 + 2r_i s_0 - 2r_0 r_i s_0 - s_i + {r_0}^2 s_i + r_0 s_i - {r_0}^3 s_i - r_i s_0 + {r_0}^2 r_i s_0]$$

Collect similar terms

$$M_i = \frac{1}{\sqrt{2H}}\frac{1}{(1-r_0)^2}[-r_0 s_i + 2{r_0}^2 s_i + r_i s_0 - 2r_0 r_i s_0 + {r_0}^2 r_i s_0 - {r_0}^3 s_i]$$

$$M_i = \frac{1}{\sqrt{2H}}\frac{1}{(1-r_0)^2}[-r_0 s_i(1-2r_0+{r_0}^2) + r_i s_0(1-2r_0+{r_0}^2)]$$

$$M_i = -\frac{1}{\sqrt{2H}}(r_0 s_i - r_i s_0)$$

Substitute from mapping *G₂*, equations (59) through (62)

$$M_i = -\frac{1}{\sqrt{2H}}\left[\left(\xi_0 cosh(\varphi) + \frac{\eta_0}{\sqrt{-\eta^2}}sinh(\varphi)\right)\left(-\xi_i sinh(\varphi) - \frac{\eta_k}{\sqrt{-\eta^2}}cosh(\varphi)\right) - \left(\xi_i cosh(\varphi) + \frac{\eta_i}{\sqrt{-\eta^2}}sinh(\varphi)\right)\left(-\xi_0 sinh(\varphi) - \frac{\eta_0}{\sqrt{-\eta^2}}cosh(\varphi)\right)\right]$$

Expand

$$M_i = -\frac{1}{\sqrt{2H}}\left(-\xi_0\xi_i cosh(\varphi)sinh(\varphi) - \frac{\xi_0\eta_i}{\sqrt{-\eta^2}}cosh^2(\varphi) - \frac{\xi_i\eta_0}{\sqrt{-\eta^2}}sinh^2(\varphi) + \frac{\eta_0\eta_i}{\eta^2}cosh(\varphi)sinh(\varphi) + \xi_0\xi_i cosh(\varphi)sinh(\varphi) + \frac{\xi_i\eta_0}{\sqrt{-\eta^2}}cosh^2(\varphi) + \frac{\xi_0\eta_i}{\sqrt{-\eta^2}}sinh^2(\varphi) - \frac{\eta_0\eta_i}{\eta^2}cosh(\varphi)sinh(\varphi)\right)$$

Collect similar terms

$$M_i = \frac{1}{\sqrt{2H}}\left(\frac{\xi_0\eta_i}{\sqrt{-\eta^2}} - \frac{\xi_i\eta_0}{\sqrt{-\eta^2}}\right)\left(cosh^2(\varphi) - sinh^2(\varphi)\right)$$

Use the previous identity of $\eta^2 = -\frac{1}{2H}$ and trigonometric identity

$$M_i = (\xi_0\eta_i - \xi_i\eta_0)$$

The next identity, equation (78), is just a restatement of the previous identity for η^2.

Continue with the Hamiltonian of the rotating Kepler problem (79). This is just a combination of the previous identities for the Kepler Hamiltonian and the angular momentum and requires no further calculation. This completes the proof of Proposition 6. ■

Applications

Investigate Kepler function

Our numerical solution is comprised of the two programs that were used to create S2 Fig. The MATLAB App Designer program **showPhi.mlapp** allows the user to choose values for $\boldsymbol{x}$ and $\boldsymbol{y}$, and then plots φ (blue line), $xsin(\varphi) - ycos(\varphi)$ (green line), and their intersection (red circle). The MATLAB script **analyzeKF.m** shows the values of the Kepler function in two different orientations.

Calculate Orbits and Time of Flight

We provide software that accepts the coordinates $\boldsymbol{p}$ and $\boldsymbol{q}$ in phase space and calculates coordinates on the cotangent bundle of the sphere (after the LS mapping), the anomalies, Kepler elements, and Delaunay variables. It also calculates orbits, including time-of-flight data, by solving the Delaunay Hamiltonian on the cotangent bundle of the sphere and using the inverse LS mapping to convert the result to normal phase space. This last mapping implicitly solves the Kepler equation.

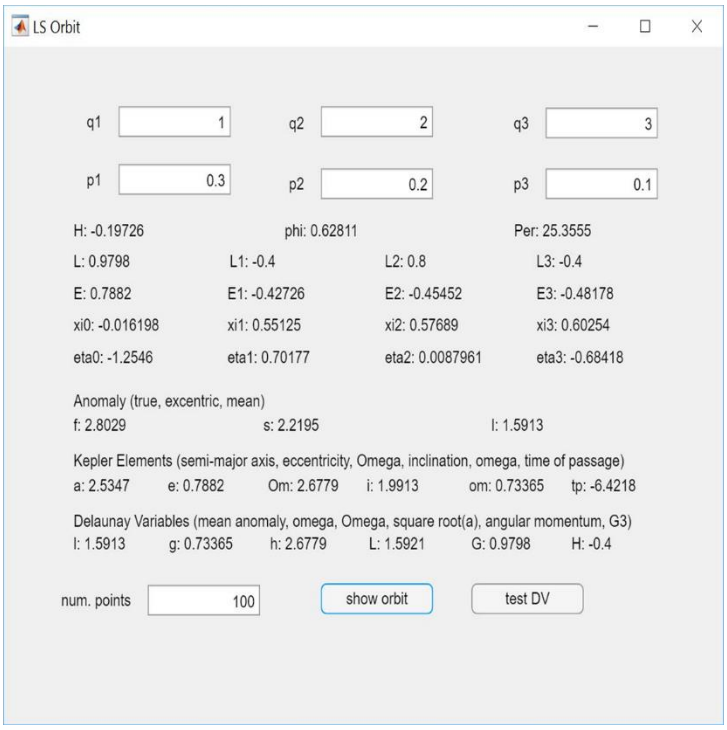

S1 Fig. LS Orbit software. The application accepts values for $\boldsymbol{q}$ and $\boldsymbol{p}$ and calculates all others.

The button **show orbit** was used to create the images in S1 Fig.

The MATLAB App Designer program **LSOrbit.mlapp** accepts values for $\boldsymbol{q}$ and $\boldsymbol{p}$ and calculates basic values (including ξ and η), anomalies, Kepler elements, and Delaunay variables (see details below). The button **show orbit** creates a time vector of "num. points" and, for all points, converts them using the forward mapping, uses the Delaunay flow to calculate the orbit based on time t, and then converts back to phase space using the inverse mapping. Then it displays the orbit and the Delaunay variables for all points of the orbit. The button **testDV** creates "num. points" points based on $\boldsymbol{q}$, $\boldsymbol{q}/2$, $\boldsymbol{q}/4$ etc., and calculates and displays the Delaunay variables for those points.

The calculations are based on the following formulas:
LS forward mapping F: equation (1) – (6).
LS inverse mapping G: equations (17) – (19).
Delaunay flow: Cushman & Bates[1] chapter 4 equation (77).
Basics

$$q = \sqrt{\sum_{i=1}^{3} q_i^2} \tag{s28}$$

$$p = \sqrt{\sum_{i=1}^{3} p_i^2} \tag{s29}$$

$$\boldsymbol{q} \cdot \boldsymbol{p} = \sum_{i=1}^{3} q_i p_i \tag{s30}$$

$$\boldsymbol{L} = \boldsymbol{q} \times \boldsymbol{p} \tag{s31}$$

Angular momentum G, Cordani[2] pg. 22.

$$L = \sqrt{\sum_{i=1}^{3} L_i^2} \tag{s32}$$

$$\boldsymbol{E} = \boldsymbol{p} \times \boldsymbol{L} - \frac{\boldsymbol{q}}{q} = -\boldsymbol{p}(\boldsymbol{q} \cdot \boldsymbol{p}) + \boldsymbol{q}p^2 - \frac{\boldsymbol{q}}{q} \tag{s33}$$

Eccentricity vector, Cordani[2] pg. 22.

$$E = \sqrt{\sum_{i=1}^{3} E_i^2} \quad \text{(s34)}$$

Eccentricity, Cordani[2] pg. 30.

$$H = \frac{p^2}{2} - \frac{1}{q} \quad \text{(s35)}$$

Hamiltonian, Cordani[2] pg. 22.
For $H<0$:

$$\varphi = \sqrt{-2H}\, \boldsymbol{q} \cdot \boldsymbol{p} \quad \text{(s36)}$$

$$\mathcal{T} = period = 2\pi/(-2H)^{3/2} \quad \text{(s37)}$$

Anomaly

$$f = cos^{-1}\left(\frac{\boldsymbol{E} \cdot \boldsymbol{q}}{Eq}\right) = cos^{-1}\left(\frac{L^2 - q}{Eq}\right) \quad \text{(s38)}$$

f is **true anomaly**, Cordani[2] pg. 23.

$$s = cos^{-1}\left(\frac{cosf + E}{Ecosf + 1}\right) \quad \text{(s39)}$$

s is **eccentric anomaly**, Cordani[2] pg. 27.

$$l = s - Esin(s) \quad \text{(s40)}$$

l is **mean anomaly**, Cordani[2] pg. 25.
Kepler Elements

$$a = \frac{1}{-2H} \quad \text{(s41)}$$

a is **semimajor axis**, Cordani[2] pg. 30-31
E is **eccentricity**, from above, Cordani[2] pg. 30.

$$\Omega = -\frac{L_2}{\sqrt{L_1^2 + L_2^2}} \quad \text{(s42)}$$

Ω is **longitude of ascending node**, Cordani[2] 30-32.

$$i = cos^{-1}\left(\frac{L_3}{L}\right) \quad \text{(s43)}$$

i is **inclination**, Cordani[2] pg. 30-32.

$$\omega = cos^{-1}\left(\frac{\boldsymbol{n} \cdot \boldsymbol{E}}{E}\right) \quad \text{(s44)}$$

ω is **argument of pericenter**, Cordani[2] pg. 31-32.

$$t_p = -a^{3/2}\big(s_0 - Esin(s_0)\big) \quad \text{(s45)}$$

t_p is **time of passage at pericenter**, Cordani[2] pg. 31-32.
Delaunay Variables

$$\ell = s - Esin(s) \quad \text{(s46)}$$

$$g = \omega \quad \text{(s47)}$$

$$h = \Omega \quad \text{(s48)}$$

$$\mathfrak{L} = \sqrt{a} = \frac{1}{\sqrt{-2H}} \quad \text{(s49)}$$

$$\mathfrak{G} = L \quad \text{(s50)}$$

$$\mathfrak{H} = L_3 \tag{s51}$$

Birkhoff conjecture for the circular restricted 3-body problem

Proof of Proposition 8. For the Kepler Hamiltonian, these expressions can be calculated in closed form.
The Hamiltonian:

$$H_K = -\frac{1}{2(\|w\|^2 + \|z\|^2)^2}$$

The gradient of the Hamiltonian:

$$\mathrm{G}_K = \nabla H_K = \frac{2}{(\|w\|^2 + \|z\|^2)^3}\begin{pmatrix} w_1 \\ w_2 \\ z_1 \\ z_2 \end{pmatrix}$$

The Hessian of the Hamiltonian:

$$Hess(H_K) = \frac{2}{(\|w\|^2 + \|z\|^2)^3}\begin{pmatrix} 1 & 0 & 0 & 0 \\ 0 & 1 & 0 & 0 \\ 0 & 0 & 1 & 0 \\ 0 & 0 & 0 & 1 \end{pmatrix} - \frac{12}{(\|w\|^2 + \|z\|^2)^4}\begin{pmatrix} w_1^2 & w_1w_2 & w_1z_1 & w_1z_2 \\ w_1w_2 & w_2^2 & w_2z_1 & w_2z_2 \\ w_1z_1 & w_2z_1 & z_1^2 & z_1z_2 \\ w_1z_2 & w_2z_2 & z_1z_2 & z_2^2 \end{pmatrix}$$

The tangential component of the gradient:

$$G_{Kt} = \frac{2}{(\|w\|^2 + \|z\|^2)^3}\begin{pmatrix} -w_2 & -z_1 & -z_2 \\ w_1 & -z_2 & z_1 \\ z_2 & w_1 & -w_2 \\ -z_1 & w_2 & w_1 \end{pmatrix}$$

The curvature on the tangential hyperspace:

$$C_{Kt} = det\left(G_{Kt}{}^T * Hess(H_K) * G_{Kt}\right) = \frac{512}{(\|w\|^2 + \|z\|^2)^{24}}$$

We'll calculate that last expression.

$$G_{Kt}{}^T * Hess(H) * G_{Kt}$$

$$= \frac{8}{(\|w\|^2 + \|z\|^2)^9}\begin{pmatrix} -w_2 & w_1 & z_2 & -z_1 \\ -z_1 & -z_2 & w_1 & w_2 \\ -z_2 & z_1 & -w_2 & w_1 \end{pmatrix}\begin{pmatrix} -w_2 & -z_1 & -z_2 \\ w_1 & -z_2 & z_1 \\ z_2 & w_1 & -w_2 \\ -z_1 & w_2 & w_1 \end{pmatrix}$$

$$- \frac{48}{(\|w\|^2 + \|z\|^2)^{10}}\begin{pmatrix} -w_2 & w_1 & z_2 & -z_1 \\ -z_1 & -z_2 & w_1 & w_2 \\ -z_2 & z_1 & -w_2 & w_1 \end{pmatrix}\begin{pmatrix} w_1^2 & w_1w_2 & w_1z_1 & w_1z_2 \\ w_1w_2 & w_2^2 & w_2z_1 & w_2z_2 \\ w_1z_1 & w_2z_1 & z_1^2 & z_1z_2 \\ w_1z_2 & w_2z_2 & z_1z_2 & z_2^2 \end{pmatrix}\begin{pmatrix} -w_2 & -z_1 & -z_2 \\ w_1 & -z_2 & z_1 \\ z_2 & w_1 & -w_2 \\ -z_1 & w_2 & w_1 \end{pmatrix}$$

$$= \frac{8}{(\|w\|^2 + \|z\|^2)^8}\begin{pmatrix} 1 & 0 & 0 \\ 0 & 1 & 0 \\ 0 & 0 & 1 \end{pmatrix} - \frac{48}{(\|w\|^2 + \|z\|^2)^{10}}\begin{pmatrix} -w_2 & w_1 & z_2 & -z_1 \\ -z_1 & -z_2 & w_1 & w_2 \\ -z_2 & z_1 & -w_2 & w_1 \end{pmatrix}\begin{pmatrix} 0 & 0 & 0 \\ 0 & 0 & 0 \\ 0 & 0 & 0 \\ 0 & 0 & 0 \end{pmatrix}$$

$$= \frac{8}{(\|w\|^2 + \|z\|^2)^8}\begin{pmatrix} 1 & 0 & 0 \\ 0 & 1 & 0 \\ 0 & 0 & 1 \end{pmatrix} - \frac{48}{(\|w\|^2 + \|z\|^2)^{10}}\begin{pmatrix} 0 & 0 & 0 \\ 0 & 0 & 0 \\ 0 & 0 & 0 \end{pmatrix}$$

$$= \frac{8}{(\|w\|^2 + \|z\|^2)^8}\begin{pmatrix} 1 & 0 & 0 \\ 0 & 1 & 0 \\ 0 & 0 & 1 \end{pmatrix}$$

$$C_{Kt} = det\left(G_{Kt}{}^{T} * Hess(H_K) * G_{Kt}\right) = \frac{512}{(\|w\|^2 + \|z\|^2)^{24}}$$

This completes the proof of Proposition 8. ■

Proof of Proposition 9. (i) Calculation of C_{Rt}.
For the planar rotating Kepler problem, these expressions can also be calculated in closed form. The Hamiltonian:

$$H_R = -\frac{1}{2(\|w\|^2 + \|z\|^2)^2} + 2(w_1 z_2 - w_2 z_1)$$

The gradient of the Hamiltonian:

$$\mathrm{G}_R = \nabla H_R = \frac{2}{(\|w\|^2 + \|z\|^2)^3}\begin{pmatrix} w_1 \\ w_2 \\ z_1 \\ z_2 \end{pmatrix} + 2\begin{pmatrix} z_2 \\ -z_1 \\ -w_2 \\ w_1 \end{pmatrix}$$

The Hessian of the Hamiltonian:

$$Hess(H_R) = \frac{2}{(\|w\|^2 + \|z\|^2)^3}\begin{pmatrix} 1 & 0 & 0 & 0 \\ 0 & 1 & 0 & 0 \\ 0 & 0 & 1 & 0 \\ 0 & 0 & 0 & 1 \end{pmatrix} - \frac{12}{(\|w\|^2 + \|z\|^2)^4}\begin{pmatrix} w_1^2 & w_1 w_2 & w_1 z_1 & w_1 z_2 \\ w_1 w_2 & w_2^2 & w_2 z_1 & w_2 z_2 \\ w_1 z_1 & w_2 z_1 & z_1^2 & z_1 z_2 \\ w_1 z_2 & w_2 z_2 & z_1 z_2 & z_2^2 \end{pmatrix}$$
$$+ 2\begin{pmatrix} 0 & 0 & 0 & 1 \\ 0 & 0 & -1 & 0 \\ 0 & -1 & 0 & 0 \\ 1 & 0 & 0 & 0 \end{pmatrix}$$

The tangential component of the gradient:

$$G_{Rt} = \frac{2}{(\|w\|^2 + \|z\|^2)^3}\begin{pmatrix} -w_2 & -z_1 & -z_2 \\ w_1 & -z_2 & z_1 \\ z_2 & w_1 & -w_2 \\ -z_1 & w_2 & w_1 \end{pmatrix} + 2\begin{pmatrix} z_1 & w_2 & -w_1 \\ z_2 & -w_1 & -w_2 \\ w_1 & z_2 & z_1 \\ w_2 & -z_1 & z_2 \end{pmatrix}$$

The curvature tangential to the hyperspace:

$$C_{Rt} = det\left(G_{Rt}{}^{T} * Hess(H_R) * G_{Rt}\right) = \text{see below}$$

In order to calculate the last expression, we'll begin by introducing some new notation, which consists of giving a name to each of the 2 terms of the gradient and the 3 terms of the Hessian. That results in a total of 12 terms for the product $G_{Rt}{}^{T} * Hess(H_R) * G_{Rt}$. Then we calculate each of these 12 terms singly, simplify and combine them, and finally calculate the determinant.

$$G_a = \begin{pmatrix} -w_2 & -z_1 & -z_2 \\ w_1 & -z_2 & z_1 \\ z_2 & w_1 & -w_2 \\ -z_1 & w_2 & w_1 \end{pmatrix}$$

$$G_a{}^{T} = \begin{pmatrix} -w_2 & w_1 & z_2 & -z_1 \\ -z_1 & -z_2 & w_1 & w_2 \\ -z_2 & z_1 & -w_2 & w_1 \end{pmatrix}$$

$$\mathrm{G}_b = \begin{pmatrix} z_1 & w_2 & -w_1 \\ z_2 & -w_1 & -w_2 \\ w_1 & z_2 & z_1 \\ w_2 & -z_1 & z_2 \end{pmatrix}$$

$$\mathrm{G}_b{}^{T} = \begin{pmatrix} z_1 & z_2 & w_1 & w_2 \\ w_2 & -w_1 & z_2 & -z_1 \\ -w_1 & -w_2 & z_1 & z_2 \end{pmatrix}$$

$$H_a = \begin{pmatrix} 1 & 0 & 0 & 0 \\ 0 & 1 & 0 & 0 \\ 0 & 0 & 1 & 0 \\ 0 & 0 & 0 & 1 \end{pmatrix}$$

$$H_b = \begin{pmatrix} {w_1}^2 & w_1w_2 & w_1z_1 & w_1z_2 \\ w_1w_2 & {w_2}^2 & w_2z_1 & w_2z_2 \\ w_1z_1 & w_2z_1 & {z_1}^2 & z_1z_2 \\ w_1z_2 & w_2z_2 & z_1z_2 & {z_2}^2 \end{pmatrix}$$

$$H_c = \begin{pmatrix} 0 & 0 & 0 & 1 \\ 0 & 0 & -1 & 0 \\ 0 & -1 & 0 & 0 \\ 1 & 0 & 0 & 0 \end{pmatrix}$$

Now we define P_{ijk} as the product of 3 corresponding terms, e.g.

$$P_{acb} = {G_a}^T H_c G_b$$

Then we define 4 more abbreviations that will be used to simplify the calculations.

$$L = 2(w_1z_2 - w_2z_1)$$

$$X = w_1^2 + w_2^2 + z_1^2 + z_2^2$$

$$Y = w_1^2 + w_2^2 - z_1^2 - z_2^2$$

$$Z = w_1z_1 + w_2z_2$$

Together, we have the following expressions:

$${G_{Rt}}^T = \frac{2}{X^3}{G_a}^T + 2{G_b}^T$$

$$Hess(H_R) = \frac{2}{X^3}H_a - \frac{12}{X^4}H_b + 2H_c$$

$$G_{Rt} = \frac{2}{X^3}G_a + 2G_b$$

Now we calculate each of the 12 terms.

$$P_{aaa} = {G_a}^T H_a G_a = \begin{pmatrix} -w_2 & w_1 & z_2 & -z_1 \\ -z_1 & -z_2 & w_1 & w_2 \\ -z_2 & z_1 & -w_2 & w_1 \end{pmatrix} \begin{pmatrix} 1 & 0 & 0 & 0 \\ 0 & 1 & 0 & 0 \\ 0 & 0 & 1 & 0 \\ 0 & 0 & 0 & 1 \end{pmatrix} \begin{pmatrix} -w_2 & -z_1 & -z_2 \\ w_1 & -z_2 & z_1 \\ z_2 & w_1 & -w_2 \\ -z_1 & w_2 & w_1 \end{pmatrix}$$

$$P_{aaa} = \begin{pmatrix} -w_2 & w_1 & z_2 & -z_1 \\ -z_1 & -z_2 & w_1 & w_2 \\ -z_2 & z_1 & -w_2 & w_1 \end{pmatrix} \begin{pmatrix} -w_2 & -z_1 & -z_2 \\ w_1 & -z_2 & z_1 \\ z_2 & w_1 & -w_2 \\ -z_1 & w_2 & w_1 \end{pmatrix}$$

$$P_{aaa} = \begin{pmatrix} w_1^2 + w_2^2 + z_1^2 + z_2^2 & 0 & 0 \\ 0 & w_1^2 + w_2^2 + z_1^2 + z_2^2 & 0 \\ 0 & 0 & w_1^2 + w_2^2 + z_1^2 + z_2^2 \end{pmatrix}$$

$$P_{aaa} = X \begin{pmatrix} 1 & 0 & 0 \\ 0 & 1 & 0 \\ 0 & 0 & 1 \end{pmatrix}$$

$$P_{aab} = {G_a}^T H_a G_b = \begin{pmatrix} -w_2 & w_1 & z_2 & -z_1 \\ -z_1 & -z_2 & w_1 & w_2 \\ -z_2 & z_1 & -w_2 & w_1 \end{pmatrix} \begin{pmatrix} 1 & 0 & 0 & 0 \\ 0 & 1 & 0 & 0 \\ 0 & 0 & 1 & 0 \\ 0 & 0 & 0 & 1 \end{pmatrix} \begin{pmatrix} z_1 & w_2 & -w_1 \\ z_2 & -w_1 & -w_2 \\ w_1 & z_2 & z_1 \\ w_2 & -z_1 & z_2 \end{pmatrix}$$

$$P_{aab} = \begin{pmatrix} -w_2 & w_1 & z_2 & -z_1 \\ -z_1 & -z_2 & w_1 & w_2 \\ -z_2 & z_1 & -w_2 & w_1 \end{pmatrix} \begin{pmatrix} z_1 & w_2 & -w_1 \\ z_2 & -w_1 & -w_2 \\ w_1 & z_2 & z_1 \\ w_2 & -z_1 & z_2 \end{pmatrix}$$

$$P_{aab} = \begin{pmatrix} 2(w_1 z_2 - w_2 z_1) & -w_1^2 - w_2^2 + z_1^2 + z_2^2 & 0 \\ w_1^2 + w_2^2 - z_1^2 - z_2^2 & 2(w_1 z_2 - w_2 z_1) & 2(w_1 z_1 + w_2 z_2) \\ 0 & -2(w_1 z_1 + w_2 z_2) & 2(w_1 z_2 - w_2 z_1) \end{pmatrix}$$

$$P_{aab} = \begin{pmatrix} L & -Y & 0 \\ Y & L & 2Z \\ 0 & -2Z & L \end{pmatrix}$$

$$P_{aba} = G_a{}^T H_b G_a$$

$$= \begin{pmatrix} -w_2 & w_1 & z_2 & -z_1 \\ -z_1 & -z_2 & w_1 & w_2 \\ -z_2 & z_1 & -w_2 & w_1 \end{pmatrix} \begin{pmatrix} w_1^2 & w_1 w_2 & w_1 z_1 & w_1 z_2 \\ w_1 w_2 & w_2^2 & w_2 z_1 & w_2 z_2 \\ w_1 z_1 & w_2 z_1 & z_1^2 & z_1 z_2 \\ w_1 z_2 & w_2 z_2 & z_1 z_2 & z_2^2 \end{pmatrix} \begin{pmatrix} -w_2 & -z_1 & -z_2 \\ w_1 & -z_2 & z_1 \\ z_2 & w_1 & -w_2 \\ -z_1 & w_2 & w_1 \end{pmatrix}$$

$$P_{aba} = \begin{pmatrix} -w_2 & w_1 & z_2 & -z_1 \\ -z_1 & -z_2 & w_1 & w_2 \\ -z_2 & z_1 & -w_2 & w_1 \end{pmatrix} \begin{pmatrix} 0 & 0 & 0 \\ 0 & 0 & 0 \\ 0 & 0 & 0 \\ 0 & 0 & 0 \end{pmatrix}$$

$$P_{aba} = \begin{pmatrix} 0 & 0 & 0 \\ 0 & 0 & 0 \\ 0 & 0 & 0 \end{pmatrix}$$

$$P_{abb} = G_a{}^T H_b G_b$$

$$= \begin{pmatrix} -w_2 & w_1 & z_2 & -z_1 \\ -z_1 & -z_2 & w_1 & w_2 \\ -z_2 & z_1 & -w_2 & w_1 \end{pmatrix} \begin{pmatrix} w_1{}^2 & w_1 w_2 & w_1 z_1 & w_1 z_2 \\ w_1 w_2 & w_2{}^2 & w_2 z_1 & w_2 z_2 \\ w_1 z_1 & w_2 z_1 & z_1{}^2 & z_1 z_2 \\ w_1 z_2 & w_2 z_2 & z_1 z_2 & z_2{}^2 \end{pmatrix} \begin{pmatrix} z_1 & w_2 & -w_1 \\ z_2 & -w_1 & -w_2 \\ w_1 & z_2 & z_1 \\ w_2 & -z_1 & z_2 \end{pmatrix}$$

$$P_{abb} = \begin{pmatrix} -w_2 & w_1 & z_2 & -z_1 \\ -z_1 & -z_2 & w_1 & w_2 \\ -z_2 & z_1 & -w_2 & w_1 \end{pmatrix} \begin{pmatrix} 2w_1{}^2 z_1 + 2w_1 w_2 z_2 & 0 & -w_1{}^3 - w_1 w_2{}^2 + w_1 z_1{}^2 + w_1 z_2{}^2 \\ 2w_2{}^2 z_2 + 2w_1 w_2 z_1 & 0 & -w_1{}^2 w_2 - w_2{}^3 + w_2 z_1{}^2 + w_2 z_2{}^2 \\ 2w_1 z_1{}^2 + 2w_2 z_1 z_2 & 0 & -w_1{}^2 z_1 - w_2{}^2 z_1 + z_1{}^3 + z_1 z_2{}^2 \\ 2w_2 z_2{}^2 + 2w_1 z_1 z_2 & 0 & -w_1{}^2 z_2 - w_2{}^2 z_2 + z_1{}^2 z_2 + z_2{}^3 \end{pmatrix}$$

$$P_{abb} = \begin{pmatrix} 0 & 0 & 0 \\ 0 & 0 & 0 \\ 0 & 0 & 0 \end{pmatrix}$$

$$P_{aca} = G_a{}^T H_c G_a = \begin{pmatrix} -w_2 & w_1 & z_2 & -z_1 \\ -z_1 & -z_2 & w_1 & w_2 \\ -z_2 & z_1 & -w_2 & w_1 \end{pmatrix} \begin{pmatrix} 0 & 0 & 0 & 1 \\ 0 & 0 & -1 & 0 \\ 0 & -1 & 0 & 0 \\ 1 & 0 & 0 & 0 \end{pmatrix} \begin{pmatrix} -w_2 & -z_1 & -z_2 \\ w_1 & -z_2 & z_1 \\ z_2 & w_1 & -w_2 \\ -z_1 & w_2 & w_1 \end{pmatrix}$$

$$P_{aca} = \begin{pmatrix} -w_2 & w_1 & z_2 & -z_1 \\ -z_1 & -z_2 & w_1 & w_2 \\ -z_2 & z_1 & -w_2 & w_1 \end{pmatrix} \begin{pmatrix} -z_1 & w_2 & w_1 \\ -z_2 & -w_1 & w_2 \\ -w_1 & z_2 & -z_1 \\ -w_2 & -z_1 & -z_2 \end{pmatrix}$$

$$P_{aca} = \begin{pmatrix} -2(w_1 z_2 - w_2 z_1) & -w_1^2 - w_2^2 + z_1^2 + z_2^2 & 0 \\ -w_1^2 - w_2^2 + z_1^2 + z_2^2 & 2(w_1 z_2 - w_2 z_1) & -2(w_1 z_1 + w_2 z_2) \\ 0 & -2(w_1 z_1 + w_2 z_2) & -2(w_1 z_2 - w_2 z_1) \end{pmatrix}$$

$$P_{aca} = \begin{pmatrix} -L & -Y & 0 \\ -Y & L & -2Z \\ 0 & -2Z & -L \end{pmatrix}$$

$$P_{acb} = G_a{}^T H_c G_b = \begin{pmatrix} -w_2 & w_1 & z_2 & -z_1 \\ -z_1 & -z_2 & w_1 & w_2 \\ -z_2 & z_1 & -w_2 & w_1 \end{pmatrix} \begin{pmatrix} 0 & 0 & 0 & 1 \\ 0 & 0 & -1 & 0 \\ 0 & -1 & 0 & 0 \\ 1 & 0 & 0 & 0 \end{pmatrix} \begin{pmatrix} z_1 & w_2 & -w_1 \\ z_2 & -w_1 & -w_2 \\ w_1 & z_2 & z_1 \\ w_2 & -z_1 & z_2 \end{pmatrix}$$

$$P_{acb} = \begin{pmatrix} -w_2 & w_1 & z_2 & -z_1 \\ -z_1 & -z_2 & w_1 & w_2 \\ -z_2 & z_1 & -w_2 & w_1 \end{pmatrix} \begin{pmatrix} w_2 & -z_1 & z_2 \\ -w_1 & -z_2 & -z_1 \\ -z_2 & w_1 & w_2 \\ z_1 & w_2 & -w_1 \end{pmatrix}$$

$$P_{acb} = \begin{pmatrix} -w_1^2 - w_2^2 - z_1^2 - z_2^2 & 0 & 0 \\ 0 & w_1^2 + w_2^2 + z_1^2 + z_2^2 & 0 \\ 0 & 0 & -w_1^2 - w_2^2 - z_1^2 - z_2^2 \end{pmatrix}$$

$$P_{acb} = X \begin{pmatrix} -1 & 0 & 0 \\ 0 & 1 & 0 \\ 0 & 0 & -1 \end{pmatrix}$$

$$P_{baa} = G_b{}^T H_a G_a = \begin{pmatrix} z_1 & z_2 & w_1 & w_2 \\ w_2 & -w_1 & z_2 & -z_1 \\ -w_1 & -w_2 & z_1 & z_2 \end{pmatrix} \begin{pmatrix} 1 & 0 & 0 & 0 \\ 0 & 1 & 0 & 0 \\ 0 & 0 & 1 & 0 \\ 0 & 0 & 0 & 1 \end{pmatrix} \begin{pmatrix} -w_2 & -z_1 & -z_2 \\ w_1 & -z_2 & z_1 \\ z_2 & w_1 & -w_2 \\ -z_1 & w_2 & w_1 \end{pmatrix}$$

$$P_{baa} = \begin{pmatrix} z_1 & z_2 & w_1 & w_2 \\ w_2 & -w_1 & z_2 & -z_1 \\ -w_1 & -w_2 & z_1 & z_2 \end{pmatrix} \begin{pmatrix} -w_2 & -z_1 & -z_2 \\ w_1 & -z_2 & z_1 \\ z_2 & w_1 & -w_2 \\ -z_1 & w_2 & w_1 \end{pmatrix}$$

$$P_{baa} = \begin{pmatrix} 2(w_1 z_2 - w_2 z_1) & w_1^2 + w_2^2 - z_1^2 - z_2^2 & 0 \\ -w_1^2 - w_2^2 + z_1^2 + z_2^2 & 2(w_1 z_2 - w_2 z_1) & -2(w_1 z_1 + w_2 z_2) \\ 0 & 2(w_1 z_1 + w_2 z_2) & 2(w_1 z_2 - w_2 z_1) \end{pmatrix}$$

$$P_{baa} = \begin{pmatrix} L & Y & 0 \\ -Y & L & -2Z \\ 0 & 2Z & L \end{pmatrix}$$

$$P_{bab} = G_b{}^T H_a G_b = \begin{pmatrix} z_1 & z_2 & w_1 & w_2 \\ w_2 & -w_1 & z_2 & -z_1 \\ -w_1 & -w_2 & z_1 & z_2 \end{pmatrix} \begin{pmatrix} 1 & 0 & 0 & 0 \\ 0 & 1 & 0 & 0 \\ 0 & 0 & 1 & 0 \\ 0 & 0 & 0 & 1 \end{pmatrix} \begin{pmatrix} z_1 & w_2 & -w_1 \\ z_2 & -w_1 & -w_2 \\ w_1 & z_2 & z_1 \\ w_2 & -z_1 & z_2 \end{pmatrix}$$

$$P_{bab} = \begin{pmatrix} z_1 & z_2 & w_1 & w_2 \\ w_2 & -w_1 & z_2 & -z_1 \\ -w_1 & -w_2 & z_1 & z_2 \end{pmatrix} \begin{pmatrix} z_1 & w_2 & -w_1 \\ z_2 & -w_1 & -w_2 \\ w_1 & z_2 & z_1 \\ w_2 & -z_1 & z_2 \end{pmatrix}$$

$$P_{bab} = \begin{pmatrix} w_1^2 + w_2^2 + z_1^2 + z_2^2 & 0 & 0 \\ 0 & w_1^2 + w_2^2 + z_1^2 + z_2^2 & 0 \\ 0 & 0 & w_1^2 + w_2^2 + z_1^2 + z_2^2 \end{pmatrix}$$

$$P_{bab} = X \begin{pmatrix} 1 & 0 & 0 \\ 0 & 1 & 0 \\ 0 & 0 & 1 \end{pmatrix}$$

$$P_{bba} = G_b{}^T H_b G_a$$

$$= \begin{pmatrix} z_1 & z_2 & w_1 & w_2 \\ w_2 & -w_1 & z_2 & -z_1 \\ -w_1 & -w_2 & z_1 & z_2 \end{pmatrix} \begin{pmatrix} w_1{}^2 & w_1 w_2 & w_1 z_1 & w_1 z_2 \\ w_1 w_2 & w_2{}^2 & w_2 z_1 & w_2 z_2 \\ w_1 z_1 & w_2 z_1 & z_1{}^2 & z_1 z_2 \\ w_1 z_2 & w_2 z_2 & z_1 z_2 & z_2{}^2 \end{pmatrix} \begin{pmatrix} -w_2 & -z_1 & -z_2 \\ w_1 & -z_2 & z_1 \\ z_2 & w_1 & -w_2 \\ -z_1 & w_2 & w_1 \end{pmatrix}$$

$$P_{bba} = \begin{pmatrix} z_1 & z_2 & w_1 & w_2 \\ w_2 & -w_1 & z_2 & -z_1 \\ -w_1 & -w_2 & z_1 & z_2 \end{pmatrix} \begin{pmatrix} 0 & 0 & 0 \\ 0 & 0 & 0 \\ 0 & 0 & 0 \\ 0 & 0 & 0 \end{pmatrix}$$

$$P_{bba} = \begin{pmatrix} 0 & 0 & 0 \\ 0 & 0 & 0 \\ 0 & 0 & 0 \end{pmatrix}$$

$$P_{bbb} = {G_b}^T H_b G_b$$

$$= \begin{pmatrix} z_1 & z_2 & w_1 & w_2 \\ w_2 & -w_1 & z_2 & -z_1 \\ -w_1 & -w_2 & z_1 & z_2 \end{pmatrix} \begin{pmatrix} {w_1}^2 & w_1 w_2 & w_1 z_1 & w_1 z_2 \\ w_1 w_2 & {w_2}^2 & w_2 z_1 & w_2 z_2 \\ w_1 z_1 & w_2 z_1 & {z_1}^2 & z_1 z_2 \\ w_1 z_2 & w_2 z_2 & z_1 z_2 & {z_2}^2 \end{pmatrix} \begin{pmatrix} z_1 & w_2 & -w_1 \\ z_2 & -w_1 & -w_2 \\ w_1 & z_2 & z_1 \\ w_2 & -z_1 & z_2 \end{pmatrix}$$

$$P_{bbb} = \begin{pmatrix} z_1 & z_2 & w_1 & w_2 \\ w_2 & -w_1 & z_2 & -z_1 \\ -w_1 & -w_2 & z_1 & z_2 \end{pmatrix} \begin{pmatrix} 2{w_1}^2 z_1 + 2w_1 w_2 z_2 & 0 & -{w_1}^3 - w_1 {w_2}^2 + w_1 {z_1}^2 + w_1 {z_2}^2 \\ 2{w_2}^2 z_2 + 2w_1 w_2 z_1 & 0 & -{w_1}^2 w_2 - {w_2}^3 + w_2 {z_1}^2 + w_2 {z_2}^2 \\ 2w_1 {z_1}^2 + 2w_2 z_1 z_2 & 0 & -{w_1}^2 z_1 - {w_2}^2 z_1 + {z_1}^3 + z_1 {z_2}^2 \\ 2w_2 {z_2}^2 + 2w_1 z_1 z_2 & 0 & -{w_1}^2 z_2 - {w_2}^2 z_2 + {z_1}^2 z_2 + {z_2}^3 \end{pmatrix}$$

$$P_{bbb} = \begin{pmatrix} z_1 & z_2 & w_1 & w_2 \\ w_2 & -w_1 & z_2 & -z_1 \\ -w_1 & -w_2 & z_1 & z_2 \end{pmatrix} \begin{pmatrix} 2w_1 Z & 0 & -w_1 Y \\ 2w_2 Z & 0 & -w_2 Y \\ 2z_1 Z & 0 & -z_1 Y \\ 2z_2 Z & 0 & -z_2 Y \end{pmatrix}$$

$$P_{bbb} = \begin{pmatrix} 4Z^2 & 0 & -2YZ \\ 0 & 0 & 0 \\ -2YZ & 0 & Y^2 \end{pmatrix}$$

$$P_{bca} = {G_b}^T H_c G_a = \begin{pmatrix} z_1 & z_2 & w_1 & w_2 \\ w_2 & -w_1 & z_2 & -z_1 \\ -w_1 & -w_2 & z_1 & z_2 \end{pmatrix} \begin{pmatrix} 0 & 0 & 0 & 1 \\ 0 & 0 & -1 & 0 \\ 0 & -1 & 0 & 0 \\ 1 & 0 & 0 & 0 \end{pmatrix} \begin{pmatrix} -w_2 & -z_1 & -z_2 \\ w_1 & -z_2 & z_1 \\ z_2 & w_1 & -w_2 \\ -z_1 & w_2 & w_1 \end{pmatrix}$$

$$P_{bca} = \begin{pmatrix} z_1 & z_2 & w_1 & w_2 \\ w_2 & -w_1 & z_2 & -z_1 \\ -w_1 & -w_2 & z_1 & z_2 \end{pmatrix} \begin{pmatrix} -z_1 & w_2 & w_1 \\ -z_2 & -w_1 & w_2 \\ -w_1 & z_2 & -z_1 \\ -w_2 & -z_1 & -z_2 \end{pmatrix}$$

$$P_{bca} = \begin{pmatrix} -w_1^2 - w_2^2 - z_1^2 - z_2^2 & 0 & 0 \\ 0 & w_1^2 + w_2^2 + z_1^2 + z_2^2 & 0 \\ 0 & 0 & -w_1^2 - w_2^2 - z_1^2 - z_2^2 \end{pmatrix}$$

$$P_{bca} = X \begin{pmatrix} -1 & 0 & 0 \\ 0 & 1 & 0 \\ 0 & 0 & -1 \end{pmatrix}$$

$$P_{bcb} = {G_b}^T H_c G_b = \begin{pmatrix} z_1 & z_2 & w_1 & w_2 \\ w_2 & -w_1 & z_2 & -z_1 \\ -w_1 & -w_2 & z_1 & z_2 \end{pmatrix} \begin{pmatrix} 0 & 0 & 0 & 1 \\ 0 & 0 & -1 & 0 \\ 0 & -1 & 0 & 0 \\ 1 & 0 & 0 & 0 \end{pmatrix} \begin{pmatrix} z_1 & w_2 & -w_1 \\ z_2 & -w_1 & -w_2 \\ w_1 & z_2 & z_1 \\ w_2 & -z_1 & z_2 \end{pmatrix}$$

$$P_{bcb} = \begin{pmatrix} z_1 & z_2 & w_1 & w_2 \\ w_2 & -w_1 & z_2 & -z_1 \\ -w_1 & -w_2 & z_1 & z_2 \end{pmatrix} \begin{pmatrix} w_2 & -z_1 & z_2 \\ -w_1 & -z_2 & -z_1 \\ -z_2 & w_1 & w_2 \\ z_1 & w_2 & -w_1 \end{pmatrix}$$

$$P_{bcb} = \begin{pmatrix} -2(w_1 z_2 - w_2 z_1) & w_1^2 + w_2^2 - z_1^2 - z_2^2 & 0 \\ w_1^2 + w_2^2 - z_1^2 - z_2^2 & 2(w_1 z_2 - w_2 z_1) & 2(w_1 z_1 + w_2 z_2) \\ 0 & 2(w_1 z_1 + w_2 z_2) & -2(w_1 z_2 - w_2 z_1) \end{pmatrix}$$

$$P_{bcb} = \begin{pmatrix} -L & Y & 0 \\ Y & L & 2Z \\ 0 & 2Z & -L \end{pmatrix}$$

Now we can combine the 12 terms with the correct factors.

$$\begin{aligned} P_{Rt} &= G_{Rt}{}^T * Hess(H_R) * G_{Rt} \\ &= \frac{8}{X^9}P_{aaa} + \frac{8}{X^6}P_{aab} - \frac{48}{X^{10}}P_{aba} - \frac{48}{X^7}P_{abb} + \frac{8}{X^6}P_{aca} + \frac{8}{X^3}P_{acb} + \frac{8}{X^6}P_{baa} \\ &+ \frac{8}{X^3}P_{bab} - \frac{48}{X^7}P_{bba} - \frac{48}{X^4}P_{bbb} + \frac{8}{X^3}P_{bca} + 8P_{bcb} \end{aligned}$$

$$\begin{aligned} P_{Rt} = &\frac{8}{X^9}X\begin{pmatrix} 1 & 0 & 0 \\ 0 & 1 & 0 \\ 0 & 0 & 1 \end{pmatrix} + \frac{8}{X^6}\begin{pmatrix} L & -Y & 0 \\ Y & L & 2Z \\ 0 & -2Z & L \end{pmatrix} - \frac{48}{X^{10}}\begin{pmatrix} 0 & 0 & 0 \\ 0 & 0 & 0 \\ 0 & 0 & 0 \end{pmatrix} - \frac{48}{X^7}\begin{pmatrix} 0 & 0 & 0 \\ 0 & 0 & 0 \\ 0 & 0 & 0 \end{pmatrix} \\ &+ \frac{8}{X^6}\begin{pmatrix} -L & -Y & 0 \\ -Y & L & -2Z \\ 0 & -2Z & -L \end{pmatrix} + \frac{8}{X^3}X\begin{pmatrix} -1 & 0 & 0 \\ 0 & 1 & 0 \\ 0 & 0 & -1 \end{pmatrix} + \frac{8}{X^6}\begin{pmatrix} L & Y & 0 \\ -Y & L & -2Z \\ 0 & 2Z & L \end{pmatrix} \\ &+ \frac{8}{X^3}X\begin{pmatrix} 1 & 0 & 0 \\ 0 & 1 & 0 \\ 0 & 0 & 1 \end{pmatrix} - \frac{48}{X^7}\begin{pmatrix} 0 & 0 & 0 \\ 0 & 0 & 0 \\ 0 & 0 & 0 \end{pmatrix} - \frac{48}{X^4}\begin{pmatrix} 4Z^2 & 0 & -2YZ \\ 0 & 0 & 0 \\ -2YZ & 0 & Y^2 \end{pmatrix} \\ &+ \frac{8}{X^3}X\begin{pmatrix} -1 & 0 & 0 \\ 0 & 1 & 0 \\ 0 & 0 & -1 \end{pmatrix} + 8\begin{pmatrix} -L & Y & 0 \\ Y & L & 2Z \\ 0 & 2Z & -L \end{pmatrix} \end{aligned}$$

$$\begin{aligned} P_{Rt} = &\begin{pmatrix} \frac{8}{X^8} & 0 & 0 \\ 0 & \frac{8}{X^8} & 0 \\ 0 & 0 & \frac{8}{X^8} \end{pmatrix} + \begin{pmatrix} \frac{8L}{X^6} & -\frac{8Y}{X^6} & 0 \\ \frac{8Y}{X^6} & \frac{8L}{X^6} & \frac{16Z}{X^6} \\ 0 & -\frac{16Z}{X^6} & \frac{8L}{X^6} \end{pmatrix} + \begin{pmatrix} -\frac{8L}{X^6} & -\frac{8Y}{X^6} & 0 \\ -\frac{8Y}{X^6} & \frac{8L}{X^6} & -\frac{16Z}{X^6} \\ 0 & -\frac{16Z}{X^6} & -\frac{8L}{X^6} \end{pmatrix} \\ &+ \begin{pmatrix} -\frac{8}{X^2} & 0 & 0 \\ 0 & \frac{8}{X^2} & 0 \\ 0 & 0 & -\frac{8}{X^2} \end{pmatrix} + \begin{pmatrix} \frac{8L}{X^6} & \frac{8Y}{X^6} & 0 \\ -\frac{8Y}{X^6} & \frac{8L}{X^6} & -\frac{16Z}{X^6} \\ 0 & \frac{16Z}{X^6} & \frac{8L}{X^6} \end{pmatrix} + \begin{pmatrix} \frac{8}{X^2} & 0 & 0 \\ 0 & \frac{8}{X^2} & 0 \\ 0 & 0 & \frac{8}{X^2} \end{pmatrix} \\ &+ \begin{pmatrix} -\frac{192Z^2}{X^4} & 0 & \frac{96YZ}{X^4} \\ 0 & 0 & 0 \\ \frac{96YZ}{X^4} & 0 & -\frac{48Y^2}{X^4} \end{pmatrix} + \begin{pmatrix} -\frac{8}{X^2} & 0 & 0 \\ 0 & \frac{8}{X^2} & 0 \\ 0 & 0 & -\frac{8}{X^2} \end{pmatrix} + \begin{pmatrix} -8L & 8Y & 0 \\ 8Y & 8L & 16Z \\ 0 & 16Z & -8L \end{pmatrix} \end{aligned}$$

$$P_{Rt} = \begin{pmatrix} \frac{8L}{X^6} - 8L - \frac{8}{X^2} + \frac{8}{X^8} - \frac{192Z^2}{X^4} & 8Y - \frac{8Y}{X^6} & \frac{96YZ}{X^4} \\ 8Y - \frac{8Y}{X^6} & 8L + \frac{24L}{X^6} + \frac{24}{X^2} + \frac{8}{X^8} & 16Z - \frac{16Z}{X^6} \\ \frac{96YZ}{X^4} & 16Z - \frac{16Z}{X^6} & \frac{8L}{X^6} - 8L - \frac{8}{X^2} + \frac{8}{X^8} - \frac{48Y^2}{X^4} \end{pmatrix}$$

The curvature is:

$$C_{Rt} = \det(P_{Rt})$$

$$C_{Rt} = \left(\frac{512}{X^{24}}\right)(L^3X^{24} + L^3X^{18} - 5L^3X^{12} + 3L^3X^6 + 5L^2X^{22} + 6L^2X^{20}Y^2 + 24L^2X^{20}Z^2 - 3L^2X^{16} + 12L^2X^{14}Y^2 + 48L^2X^{14}Z^2 - 9L^2X^{10} - 18L^2X^8Y^2 - 72L^2X^8Z^2 + 7L^2X^4 + LX^{24}Y^2 + 4LX^{24}Z^2 + 7LX^{20} + 21LX^{18}Y^2 + 84LX^{18}Z^2 - 9LX^{14} + 3LX^{12}Y^2 + 12LX^{12}Z^2 - 3LX^8 - 25LX^6Y^2 - 100LX^6Z^2 + 5LX^2 + X^{22}Y^2 + 4X^{22}Z^2 + 6X^{20}Y^4 + 48X^{20}Y^2Z^2 + 96X^{20}Z^4 + 3X^{18} + 15X^{16}Y^2 + 60X^{16}Z^2 - 12X^{14}Y^4 - 96X^{14}Y^2Z^2 - 192X^{14}Z^4 - 5X^{12} - 9X^{10}Y^2 - 36X^{10}Z^2 + 6X^8Y^4 + 48X^8Y^2Z^2 + 96X^8Z^4 + X^6 - 7X^4Y^2 - 28X^4Z^2 + 1)$$

(ii) Proof of positivity.
C_{Rt} can be factorized

$$C_{Rt} = \left(\frac{512}{X^{24}}\right) C_1C_2C_3C_4C_5C_6$$

$$C_1 = X - 1$$

$$C_2 = X + 1$$

$$C_3 = X^2 + X + 1$$

$$C_4 = X^2 - X + 1$$

$$C_5 = LX^8 + X^6 + 6X^4Y^2 + 24X^4Z^2 - LX^2 - 1$$

$$C_6 = L^2X^{10} + 3L^2X^4 + 4LX^8 + 4LX^2 + X^{10}Y^2 + 4X^{10}Z^2 + 3X^6 - X^4Y^2 - 4X^4Z^2 + 1$$

First, we observe that

$$Y^2 + 4Z^2 = X^2 - L^2$$

To demonstrate this, we simply expand each side and compare the results.

$$\begin{aligned} Y^2 + 4Z^2 &= (w_1^2 + w_2^2 - z_1^2 - z_2^2)^2 + 4(w_1z_1 + w_2z_2)^2 \\ &= w_1^4 + w_2^4 + z_1^4 + z_2^4 + 2w_1^2w_2^2 - 2w_1^2z_1^2 - 2w_1^2z_2^2 - 2w_2^2z_1^2 - 2w_2^2z_2^2 + 2z_1^2z_2^2 \\ &+ 4w_1^2z_1^2 + 4w_2^2z_2^2 + 8w_1w_2z_1z_2 \\ &= w_1^4 + w_2^4 + z_1^4 + z_2^4 + 2w_1^2w_2^2 + 2w_1^2z_1^2 - 2w_1^2z_2^2 - 2w_2^2z_1^2 + 2w_2^2z_2^2 + 2z_1^2z_2^2 \\ &+ 8w_1w_2z_1z_2 \end{aligned}$$

$$\begin{aligned} X^2 - L^2 &= (w_1^2 + w_2^2 + z_1^2 + z_2^2)^2 - 4(w_1z_2 - w_2z_1)^2 \\ &= w_1^4 + w_2^4 + z_1^4 + z_2^4 + 2w_1^2w_2^2 + 2w_1^2z_1^2 + 2w_1^2z_2^2 + 2w_2^2z_1^2 + 2w_2^2z_2^2 + 2z_1^2z_2^2 \\ &- 4w_1^2z_2^2 - 4w_2^2z_1^2 + 8w_1w_2z_1z_2 \\ &= w_1^4 + w_2^4 + z_1^4 + z_2^4 + 2w_1^2w_2^2 + 2w_1^2z_1^2 - 2w_1^2z_2^2 - 2w_2^2z_1^2 + 2w_2^2z_2^2 + 2z_1^2z_2^2 \\ &+ 8w_1w_2z_1z_2 \end{aligned}$$

Now we use this result to simplify C_5 and C_6.

$$\begin{aligned} C_5 &= LX^8 + X^6 + 6X^4Y^2 + 24X^4Z^2 - LX^2 - 1 = (LX^2 + 1)(X^6 - 1) + 6X^4(Y^2 + 4Z^2) \\ &= (LX^2 + 1)(X^6 - 1) + 6X^4(X^2 - L^2) \end{aligned}$$

$$C_5 = -6L^2X^4 + LX^8 - LX^2 + 7X^6 - 1$$

$$\begin{aligned} C_6 &= L^2X^{10} + 3L^2X^4 + 4LX^8 + 4LX^2 + X^{10}Y^2 + 4X^{10}Z^2 + 3X^6 - X^4Y^2 - 4X^4Z^2 + 1 \\ &= L^2X^4(X^6 + 3) + 4LX^2(X^6 + 1) + 3X^6 + 1 + X^4(Y^2 + 4Z^2)(X^6 - 1) \\ &= L^2X^4(X^6 + 3) + 4LX^2(X^6 + 1) + 3X^6 + 1 + X^4(X^2 - L^2)(X^6 - 1) \\ &= L^2X^4(X^6 + 3 - X^6 + 1) + 4LX^2(X^6 + 1) + 3X^6 + 1 + X^6(X^6 - 1) \\ &= 4L^2X^4 + 4LX^2(X^6 + 1) + X^{12} + 2X^6 + 1 \end{aligned}$$

$$C_6 = (X^6 + 2LX^2 + 1)^2$$

Together, we have:

$$C_{Rt} = \left(\frac{512}{X^{24}}\right) C_1C_2C_3C_4C_5C_6$$

$$C_1 = X - 1$$
$$C_2 = X + 1$$
$$C_3 = X^2 + X + 1$$
$$C_4 = X^2 - X + 1$$
$$C_5 = -6L^2X^4 + LX^8 - LX^2 + 7X^6 - 1$$
$$C_6 = (X^6 + 2LX^2 + 1)^2$$

$X<1$ iff $H_K<-0.5$
$C_1<0$ iff $H_K<-0.5$
$C_2>0$ always (sum of positive terms)
$C_3>0$ always (sum of positive terms)
$C_4>0$ for $H_K<-0.5$ (because $1-X>0$)
$C_5<0$ if $H_R<-1.5$ (via analytical proof assuming $|L| < X$ and $H_R<-1.5$, see below)
$C_6>0$ always (squared expression)
$C>0$ if $H_K<-0.5$, $H_R<-1.5$, and $|L| < X$.

$H_K<-0.5$ is necessary: point (0.8, 0.9, 0.9, -0.9) has $H_K>-0.5$, $H_R<-1.5$ and $C<0$ ($C1>0$, $C5<0$).
$H_R<-1.5$ is necessary: point (0.3, 0.4, 0.5, 0.5) has $H_K<-0.5$, $H_R>-1.5$ and $C<0$ ($C1<0$, $C5>0$).

As a preliminary step, we observe

$$H_K = -\frac{1}{2X^2} = -\frac{1}{2|\eta|^2} = -\frac{1}{2a}$$

where a is the semimajor axis. Then we conclude

$$H_K \leq -\frac{1}{2} \Leftrightarrow |X| \leq 1 \Leftrightarrow a \leq 1 \Leftrightarrow |\eta| \leq 1$$

By examining the ellipse, we can see that the semimajor axis $a < max(|q|)$ so, since $|q| < 1$ for all points in the bounded component of the Hill region,

$$a < 1.$$

Then we recall

$$|L| = \sqrt{a}\sqrt{1-e^2}$$

where e is the eccentricity and $0 < e < 1$ for an ellipse, and we conclude

$$|L| < 1$$
$$|L| = |X|\sqrt{1-e^2} < |X| \Rightarrow |L| < |X|.$$

altogether:

$$|L| < |X| \leq 1.$$

Now, we make some observations and calculate some special cases for C_5.
First, we state the main constraint

$$H_R = -\frac{1}{2X^2} + L \leq -\frac{3}{2}.$$

We used numerical optimization, specifically MATLAB **fmincon**, to calculate the maximum value of C_5 assuming that $0 \leq X \leq 1, -1 \leq L \leq 1$, and $H_R \leq -\frac{3}{2}$.
The result is (approximately)

$$\max(C_5) = 0 \; at \; X = 1 \; and \; L = -1.$$

If we assume that $0 \leq X \leq 1, -1 \leq L \leq 1$, but leave the other inequality out, we have

$$\max(C_5) = 6.$$

Now we proceed with the analytical proof that $C_5 \leq 0$. We begin with a number of plots that help us gain some insight, and then make all necessary calculations.

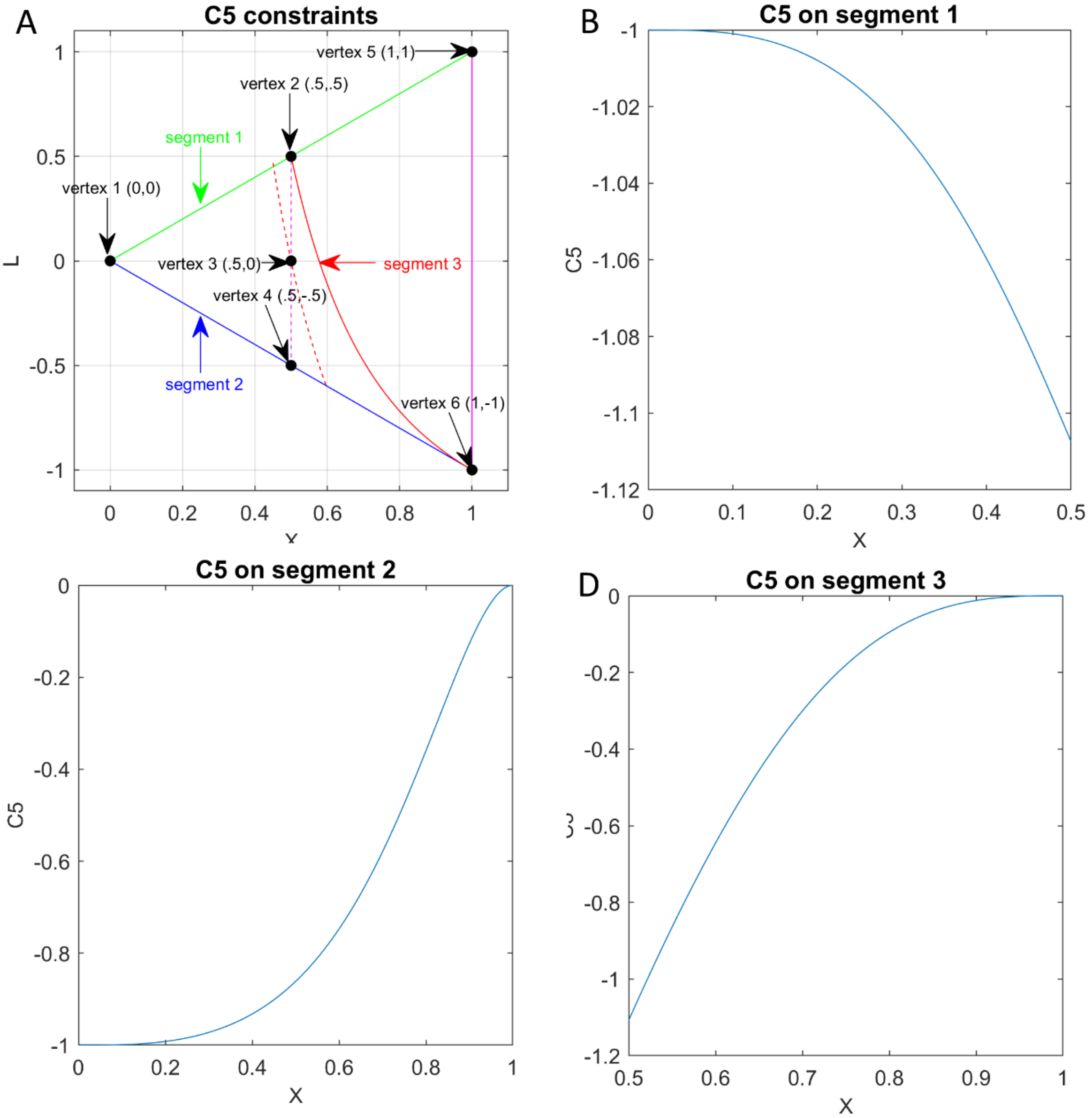

S2 Fig. C_5 constraints and plots. The plots show aspects of the factor C_5 of the curvature of the rotating Hamiltonian H_R. (A) The constraints on C_5 appear as boundaries of a region in X-L space. (B) C_5 on segment 1 ($L = X$). (C) C_5 on segment 2 ($L = -X$). (D) C_5 on segment 3 ($L = -1.5 + \frac{1}{2X^2}$).

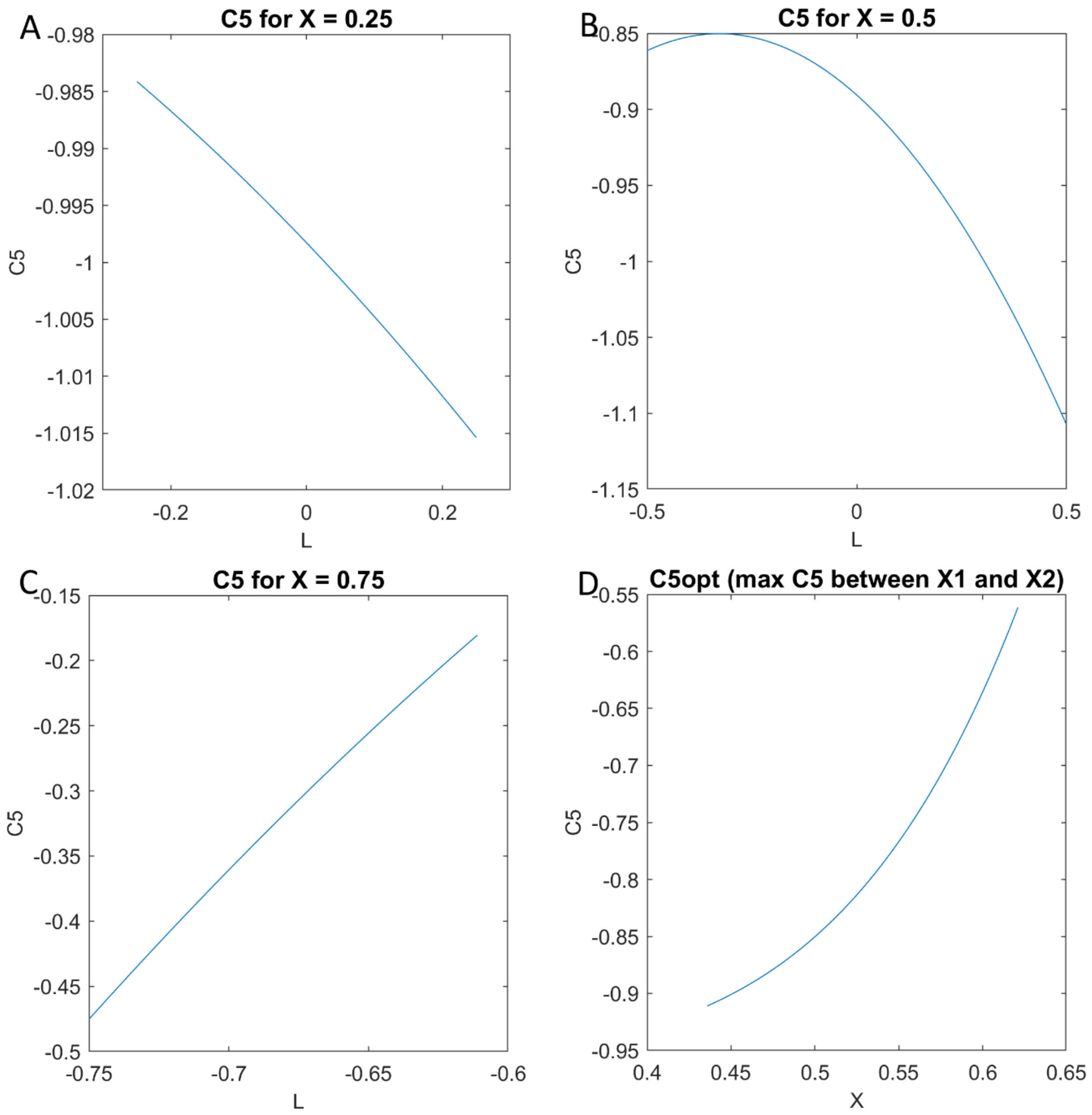

S3 Fig. C_5 plots at specific points. The plots show aspects of the factor C_5 of the curvature of the rotating Hamiltonian H_R. (A) C_5 on $X = .25$. (B) C_5 on $X = .5$. (C) C_5 on $X = .75$. (D) Maximum of C_5 for $X_1 \leq X \leq X_2$.

On segment 1,

$$C_{5s1} = C_5|_{L=X} = X^9 + X^6 - X^3 - 1 = (X-1)(X+1)^2(X^2+X+1)(X^2-X+1)^2$$

$$\frac{dC_{5s1}}{dX} = 9X^8 + 6X^5 - 3X^2 = 3X^2(X+1)(X^2-X+1)(3X^3-1) \leq 0$$

because $X \leq .5$. As a result, C_5 on segment 1 is monotonically decreasing, and:
The maximum of C_5 on segment 1 is -1 at $(X, L) = (0,0)$.

On segment 2,

$$C_{5s2} = C_5|_{L=-X} = -X^9 + X^6 + X^3 - 1 = (-1)(X+1)(X-1)^2(X^2-X+1)(X^2+X+1)^2$$

$$\frac{dC_{5s2}}{dX} = -9X^8 + 6X^5 + 3X^2 = -3X^2(X-1)(X^2+X+1)(3X^3+1) \geq 0$$

because $X \leq 1$. As a result, C_5 on segment 2 is monotonically increasing, and:
The maximum of C_5 on segment 2 is 0 at $(X, L) = (1, -1)$.

On segment 3,

$$C_{5s3} = C_5|_{L=-\frac{3}{2}+\frac{1}{2X^2}} = \frac{-3X^8}{2} + \frac{15X^6}{2} - \frac{27X^4}{2} + \frac{21X^2}{2} - 3 = -\frac{3}{2}(X-1)^3(X+1)^3(X^2-2)$$

$$\frac{dC_{5s3}}{dX} = -12X^7 + 45X^5 - 54X^3 + 21X = -3X(X+1)^2(X-1)^2(4X^2-7) \geq 0$$

because $X \leq 1$. As a result, C_5 on segment 3 is monotonically increasing, and:
The maximum of C_5 on segment 3 is 0 at $(X, L) = (1, -1)$.

Now we need to find the maximum of C_5 in the interior, and we will begin by thinking of Xas constant, as illustrated in S3 Fig B, C, and C, and taking the derivative with respect to L:

$$\frac{dC_5}{dL} = X^8 - 12LX^4 - X^2 = X^2(X^6 - 12LX^2 - 1)$$

First, we simultaneously solve the following equations for L:

$$\frac{dC_5}{dL} = 0$$

$$-\mathrm{X} \leq \mathrm{L} \leq \mathrm{X}$$

$$\mathrm{L} \leq -\frac{3}{2} + \frac{1}{2X^2}.$$

The result is

$$L_{opt} := \frac{X^6-1}{12X^2}.$$

on condition that

$$0 \leq X^2(X^6 + 12X^3 - 1)$$

$$X^2(X^6 + 18X^2 - 7) \leq 0$$

$$L_{opt} \leq X.$$

The conditions show us the limits for the resulting optimum, so we will define them as constants. First, we simultaneously solve the following equations for X:

$$X^2(X^6 + 12X^3 - 1)$$

$$-\mathrm{X} \leq \mathrm{L} \leq \mathrm{X}$$

$$\mathrm{L} \leq -\frac{3}{2} + \frac{1}{2X^2}.$$

The result is

$$X_1 := \left(\sqrt{37} - 6\right)^{1/3} \approx 0.44.$$

Then we simultaneously solve

$$X^2(X^6 + 18X^2 - 7)$$

$$-\mathrm{X} \leq \mathrm{L} \leq \mathrm{X}$$

$$\mathrm{L} \leq -\frac{3}{2} + \frac{1}{2X^2}.$$

The result is

$$X_2 := \sqrt{root(z^3 + 18z - 7, z, 1)} \approx 0.62.$$

Now we look at the second derivative

$$\frac{d^2C_5}{dL^2} = -12X^4 \leq 0,$$

which shows us that L_{opt} is a local maximum of C_5 for $X_1 \leq X \leq X_2$.Now we will check C_5 in this region:

$$C_{5\mathrm{opt}} := C_5|_{L=L_{opt}} = \frac{1}{24}(X^{12} + 166X^6 - 23)$$

and we take the derivative

$$\frac{dC_{5\mathrm{opt}}}{dX} = \frac{1}{2}(X^{11} + 83X^5) = \frac{1}{2}X^5(X^6 + 83) \geq 0$$

so $C_{5\mathrm{opt}}$ is monotonically increasing and the maximum is at the upper end of the range, so we can substitute $X_2$for Xin $C_{5\mathrm{opt}}$ and L_{opt} and find a numerical solution:
The maximum of C_5 for $X_1 \leq X \leq X_2$is approximately -0.56 at $(X, L) = (0.62, -0.20)$.

Next, we consider the case of $X \leq X_1$.
From above, we know

$$\frac{dC_5}{dL} = X^8 - 12LX^4 - X^2 = X^2(X^6 - 12LX^2 - 1)$$

Since $L \geq -X$, or $-L \leq X$ and $X \leq X_1$, we see that

$$\frac{dC_5}{dL} \leq \frac{dC_5}{dL}|_{-L=X} = X^2(X^6 + 12X^3 - 1) \leq \frac{dC_5}{dL}|_{-L=X \wedge X=X_1} = 0$$

This shows that, for $X \leq X_1$, C_5 is monotonically decreasing, so the maximum of C_5 is on segment 2, and equal to

$$C_{5s2}|_{X=X_1} = (-1)(X+1)(X-1)^2(X^2 - X + 1)(X^2 + X + 1)^2|_{X=X_1} = 948 - 156\sqrt{37} \approx -0.91$$

The maximum of C_5 for $X \leq X_1$is approximately -0.91 at $(X, L) = (0.44, -0.44)$.

Next, we consider the case of $X_2 \leq X$.
From above, we know

$$\frac{dC_5}{dL} = X^8 - 12LX^4 - X^2 = X^2(X^6 - 12LX^2 - 1)$$

Since $-L \geq \frac{3}{2} - \frac{1}{2X^2}$ and $X \geq X_2$, we see that

$$\frac{dC_5}{dL} \geq \frac{dC_5}{dL}|_{-L=\frac{3}{2}-\frac{1}{2L^2}} = X^2(X^6 + 18X^2 - 7) \geq \frac{dC_5}{dL}|_{-L=\frac{3}{2}-\frac{1}{2L^2} \wedge X=X_2} = 0$$

This shows that, for $X \geq X_2$, C_5 is monotonically increasing, so the maximum of C_5 is on segment 3, and equal to

$$C_{5s3}|_{X=1} = -\frac{3}{2}(X-1)^3(X+1)^3(X^2 - 2)|_{X=1} = 0$$

The maximum of C_5 for $X \leq X_1$is 0 at $(X, L) = (1, -1)$.
Overall, the maximum of C_5 is 0 at $(X, L) = (1, -1)$ and, as a result, $C$is always positive.
(iii) As a direct result of (ii), the energy hypersurface is convex.
This completes the proof of Proposition 9. ■

Details of Observation 10.
The calculations on a grid are done in **CCgrid.m**, and the results have been summarized in plots S4 Fig through S9 Fig. The constrained optimization is done in **CCmin.m**, and the results have

been summarized in **CCminSummary.xlsx**. **CCmin.m** uses the MATLAB routine **fmincon** for calculating a constrained minimum using the constraints that the energy is less than the energy of the Lagrange point L_1 and the distance to the heavy primary is less than the distance to L_1. Both scripts make use of multiple MATLAB subroutines for calculating Hamiltonian and curvature. The generalized Kepler equation is solved in **KF.m** (called by **LSG.m**) by the MATLAB function **vpasolve**, and the gradient and Hessian of the Hamiltonian are calculated using the package **Derivest** (Adaptive Robust Numerical Differentiation).
MATLAB: https://www.mathworks.com/products/matlab.html
Derivest: https://www.mathworks.com/matlabcentral/fileexchange/13490-adaptive-robust-numerical-differentiation
This completes the details of Observation10. ■

Discussion of Observation 10.

For Sun-Mars and Sun-Earth, S4 Fig shows the distribution of energy and curvature of the energy.

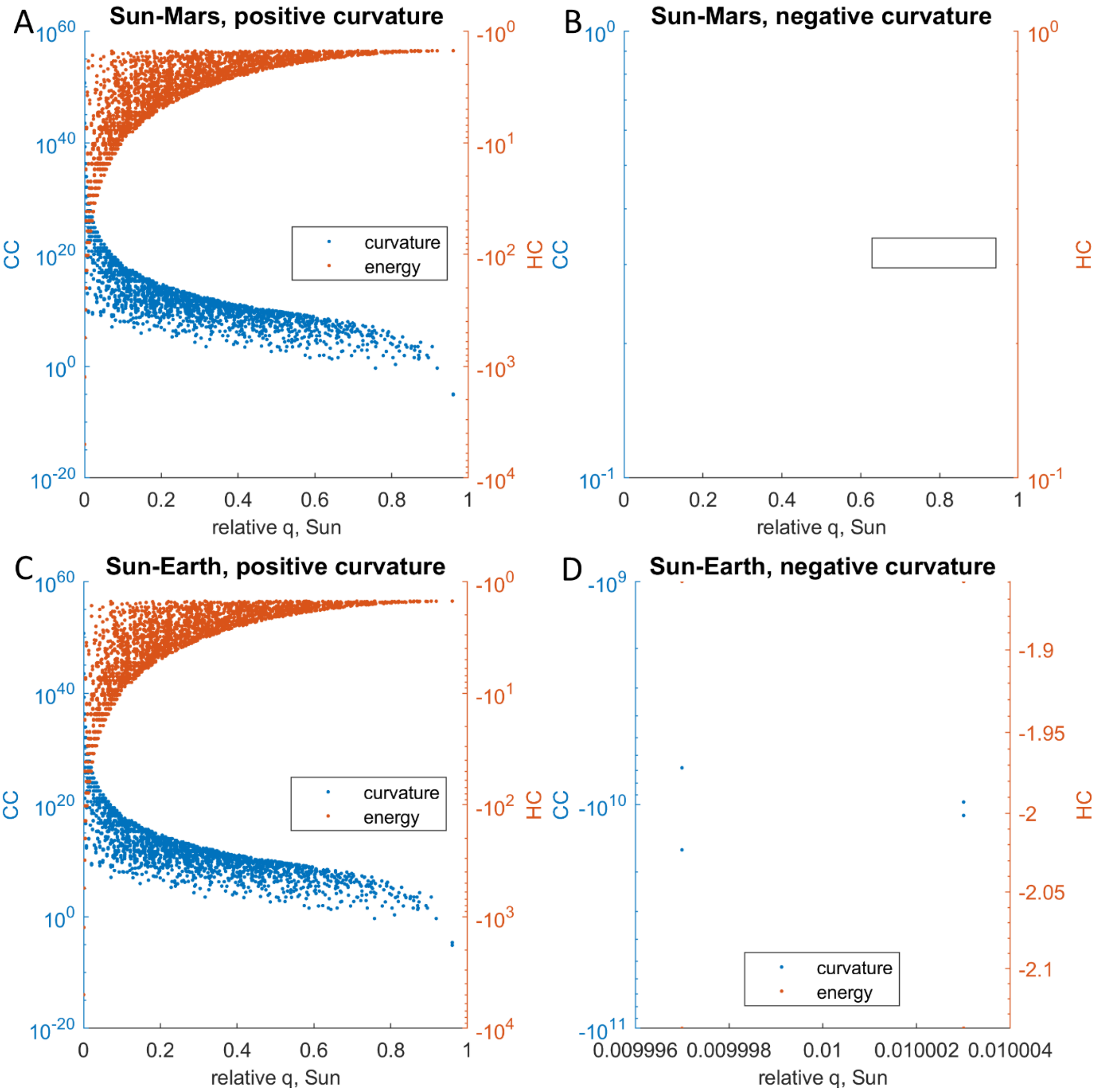

S4 Fig. Sun-Mars and Sun-Earth energy and curvature. (A) The points with positive curvature for Sun-Mars. (B) Points with negative curvature for Sun-Mars. (C) The points with positive

curvature for Sun-Earth. (D) Negative curvature for Sun-Earth. All were calculated on a grid where all four coordinates varied from -1 to 1 in intervals of 0.1.

The software **CCgrid.m** and **CCgridPlot.m** was used to create S4 Fig.

For Sun-Mars, the grid used did not find any points of negative curvature; however, our other calculations did find a few. For Sun-Earth, the grid used found a few points (8 to be exact) with negative curvature and relative distance to the Sun of less than .01.

For Sun-Jupiter, S5 Fig shows the distribution of energy and curvature of the energy, first over a wide range of points, then over a much smaller range of points centered around one point that has negative curvature.

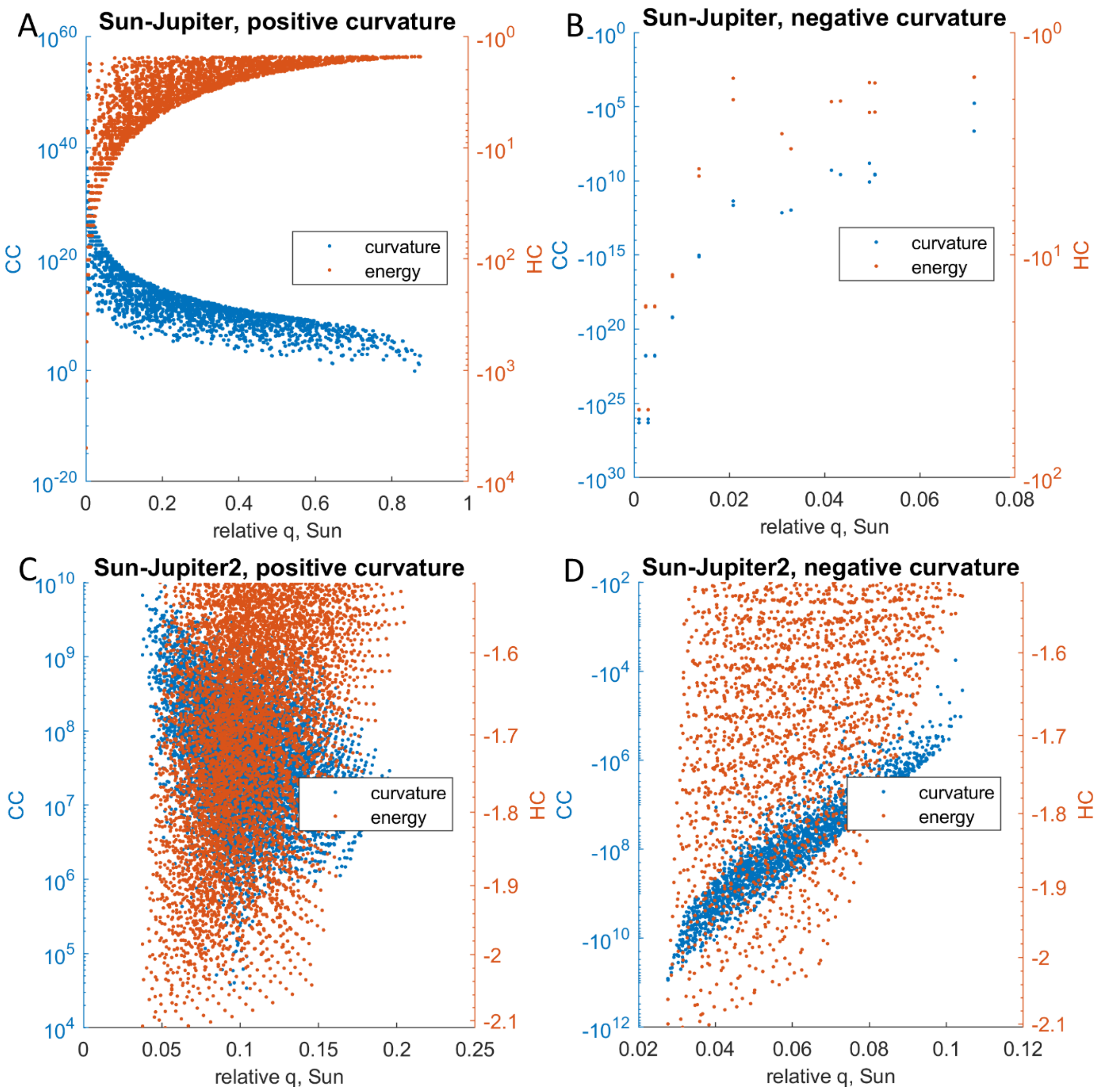

S5 Fig. Sun-Jupiter energy and curvature. (A) The points with positive curvature. (B) Points with negative curvature. (C) Positive curvature. (D) Negative curvature. (A) and (B) were calculated on a grid where all four coordinates varied from -1 to 1 in intervals of 0.1. (C) and (D) were calculated around the point (-.7,-.3,-.1,.2)±(0.05, 0.05,0.05, 0.05,) in intervals of 0.01.

The software **CCgrid.m** and **CCgridPlot.m** was used to create S5 Fig.

In S5 Fig A (points with positive curvature) and S5 Fig B (points with negative curvature), we can see that the energy (HC for circular restricted Hamiltonian, red points) is very close to the energy of the Lagrangian point L_1 when the third body is close to L_1 (q near 1). When the body is close to the heavy primary (q near 0), the potential energy can achieve large negative values, but can also approach the energy of L_1, since it can be balanced by a large kinetic energy. It appears as though this large variance of the energy also makes a negative curvature possible.

In S5 Fig C and D we show the analogous diagram but calculated on a grid of points centered around a point with negative curvature.

S6 Fig shows an example of negative curvature.

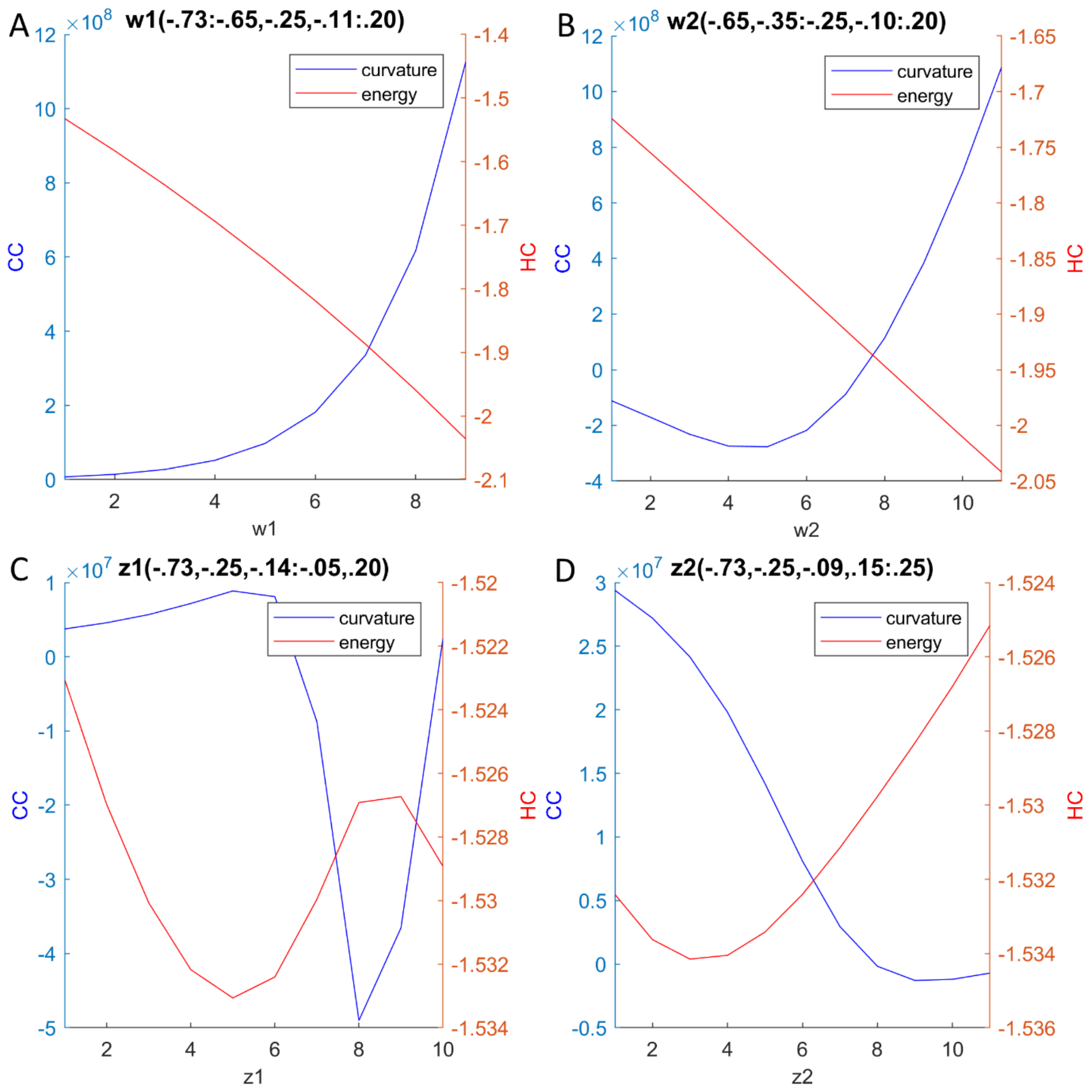

S6 Fig. Sun-Jupiter energy and curvature 2. Here we keep three coordinates fixed and vary one of them. (A), (B), (C) and (D) shows varying w1, w2, z1, and z2.

The software **CCgridSJ2.m** was used to create S6 Fig.

In S6 Fig, we show the result of keeping three coordinates fixed and varying the fourth one. S6 Fig C shows the case where the coordinate z1 was varied and displays a local maximum of the energy with a corresponding local minimum for the curvature, in this case attaining a

negative value. The diagrams are only for the purpose of exploring the possibilities and looking for patterns, since restricting to one dimension greatly reduces the information presented.

For the larger values of μ, we need to adjust the value of the energy c in order to avoid connected Hill regions, so this is plotted in S8 Fig.

For Earth-Moon S7 Fig shows the distribution of energy and curvature of the energy.

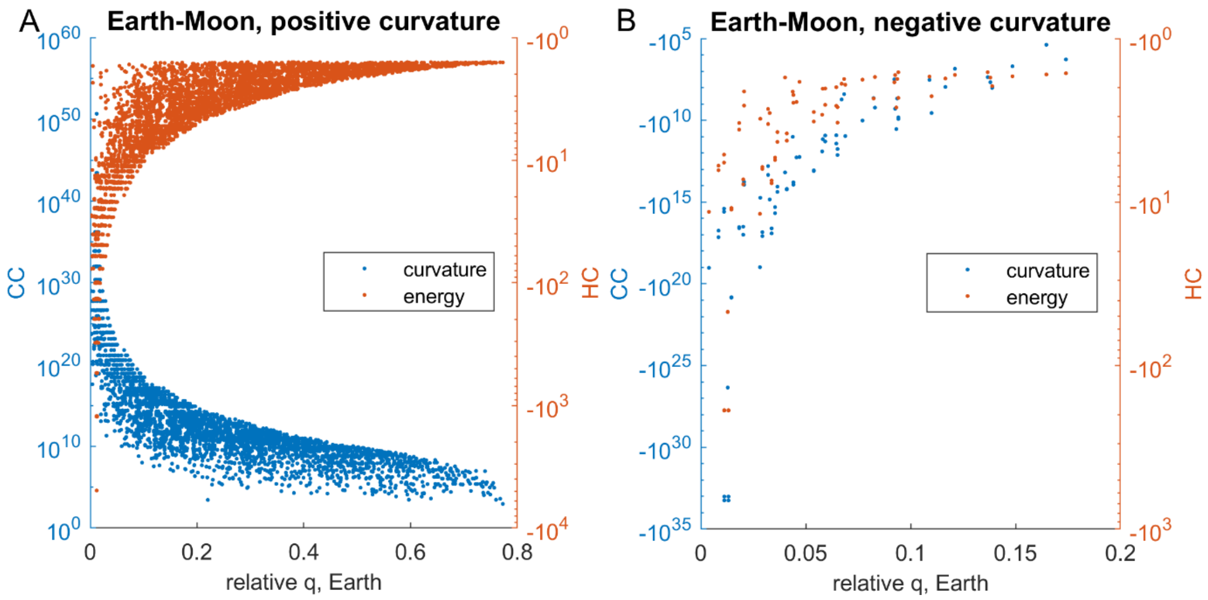

S7 Fig. Earth-Moon energy and curvature. (A) The points with positive curvature for Earth-Moon. (B) Points with negative curvature for Earth-Moon. All were calculated on a grid where all four coordinates varied from -1 to 1 in intervals of 0.1.

The software **CCgrid.m** and **CCgridPlot.m** was used to create S7 Fig.

For higher values of μ, we need to avoid connected Hill regions, so we explore this with some different values of the energy in S8 Fig.

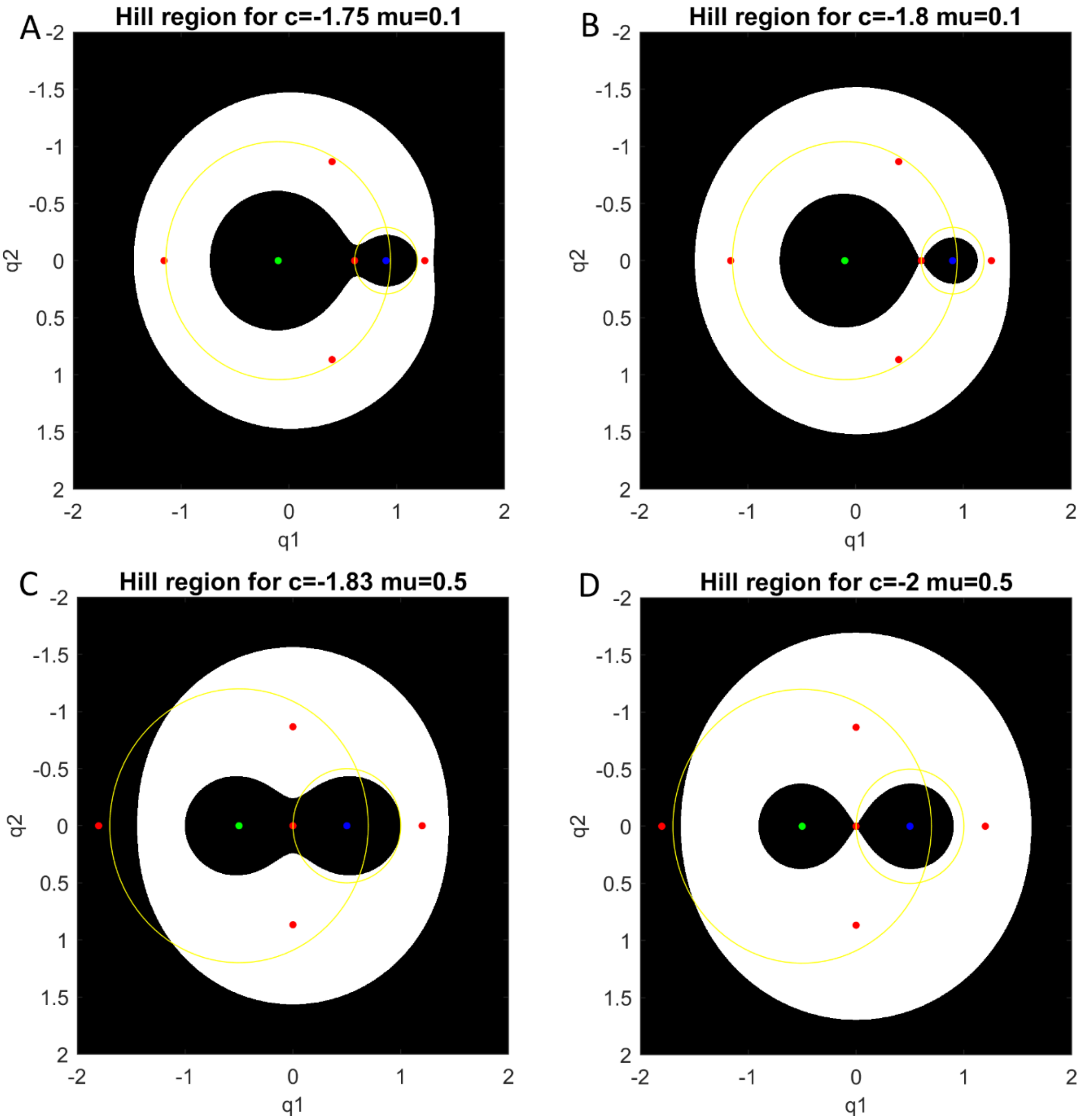

S8 Fig. Hill regions. (A), (B) Hill regions for μ=0.1. (C) and (D) Hill regions for μ=0.5. (A) and (C) use the energy of the Lagrangian point L_1, and these Hill regions are connected, so we moved to a more negative value of the energy to produce (B) and (D).

For large values of μ, S9 Fig shows the distribution of energy and curvature of the energy.

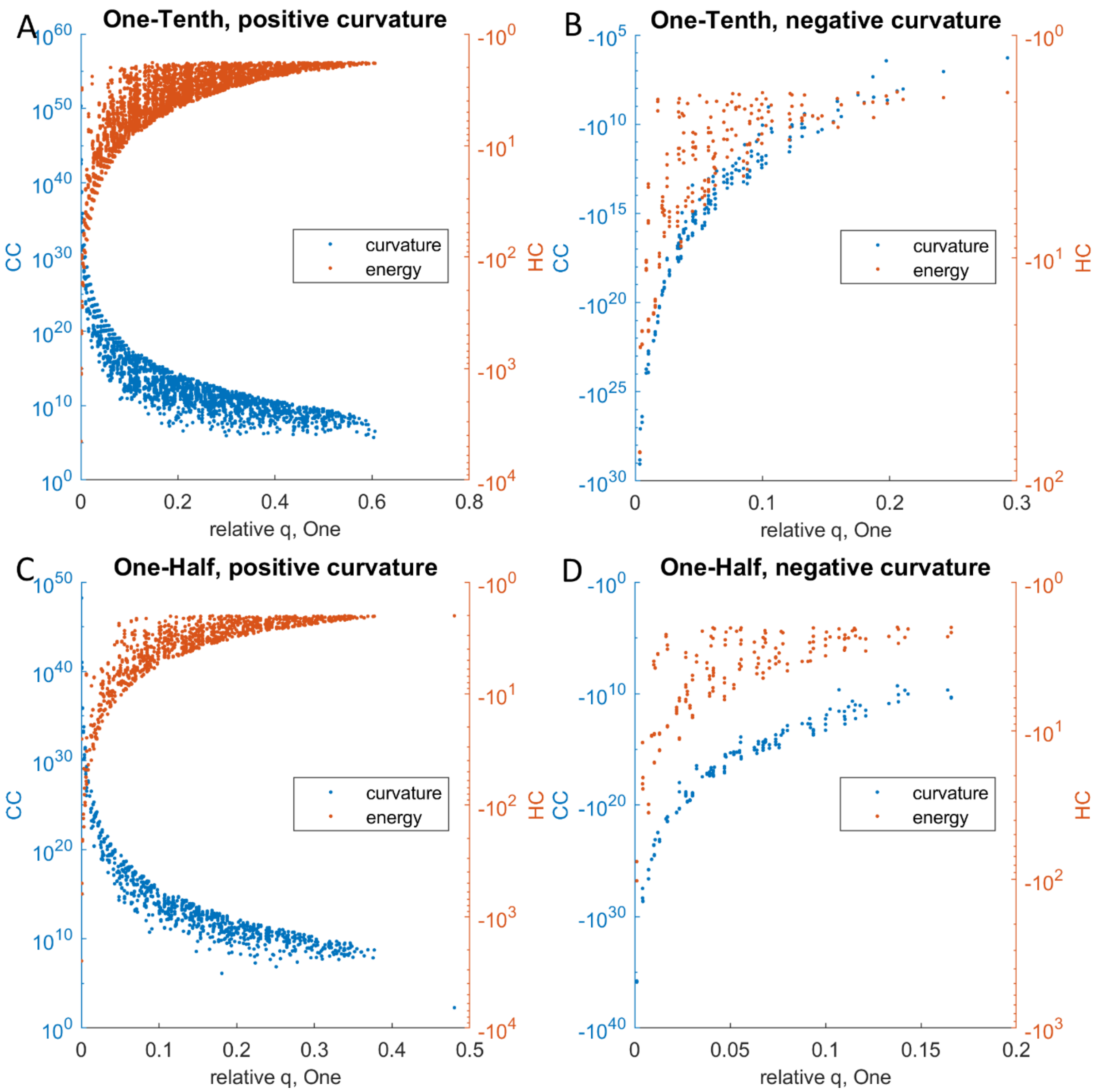

S9 Fig. $\mu = 0.1$ and $\mu = 0.5$ energy and curvature. (A) The points with positive curvature for $\mu = 0.1$ ($c = -1.8$). (B) Points with negative curvature for $\mu = 0.1$ ($c = -1.8$). (C) The points with positive curvature for $\mu = 0.5$ ($c = -2.0$). (D) Negative curvature for $\mu = 0.5$ ($c = -2.0$).. All were calculated on a grid where all four coordinates varied from -1 to 1 in intervals of 0.1.

The software **CCgrid.m** and **CCgridPlot.m** was used to create S9 Fig.

Here, we used the somewhat arbitrary values of energy c found by looking at the Hill regions in S8 Fig. We can see that the desired convexity, i.e. positive curvature, is almost totally lost for these values of μ.

Description of Software

S1_Software.zip

MATLAB software for calculations and plots. "**.m**" is a MATLAB script and can be opened in a standard text editor. Most of the code is fairly easy to read without experience in MATLAB,

except for special functions such as **fmincon** and **vpasolve**, which can be found on the Internet. "**.mlx**" is a MATLAB "live script" and requires MATLAB for opening. "**.mlapp**" is a MATLAB application and requires MATLAB app designer for opening.

- **analyzeKF.m** – analysis of the Kepler Function (generalized Kepler equation), used in Fig 2 B-C.
- **C5plot.mlx** – plots of behavior of factor C_5 in the curvature of the planar rotating (2-body) Hamiltonian, used in the proof of proposition 9.
- **CCexplore.m** – used for ad hoc calculations of the curvature of the circular restricted 3-body Hamiltonian.
- **CCgrid.m** – calculate the curvature of the circular restricted 3-body Hamiltonian on a grid of points, used in the details of observation 10.
- **CCgridPlot.m** - plot the curvature and energy of the circular restricted 3-body Hamiltonian on a grid of points, used in the discussion of observation 10.
- **CCgridSJ2.m** – similar to **CCgridPlot.m**, but for a special case of Sun-Jupiter.
- **CCmin.m** – use constrained optimization (MATLAB **fmincon**) to calculate the minimum curvature of the circular restricted 3-body Hamiltonian given a specific starting point.
- **CCSummaryPlot.m**
- **CRgrid.m** – similar to **CCgrid.m**, but for the planar rotating (2-body) Hamiltonian.
- **CRmin.m** – similar to **CCmin.m**, but for the planar rotating (2-body) Hamiltonian.
- **DF.m** – Delaunay flow, used in Fig 3.
- **HC.m** - circular restricted 3-body Hamiltonian.
- **HK.m** – Kepler Hamiltonian.
- **HR.m** - planar rotating (2-body) Hamiltonian.
- **KF.m** – Kepler Function, solves the generalized Kepler equation using MATLAB **vpasolve**.
- **LCF.m** – Levi-Civita forward mapping, see Fig 4 and following text.
- **LCG.m** – Levi-Civita inverse mapping, see Fig 4 and following text.
- **LSF.m** – Ligon-Schaaf forward mapping, see Fig 1 and following text.
- **LSG.m** – Ligon-Schaaf inverse mapping, see Fig 1 and following text, uses **KF.m**.
- **LSOrbit.mlapp** – plot orbits, using **DF.m** and **LSG.m**, used in Fig 3.
- **LSPlots.m** – plots of Kepler Function, used in Fig 2.
- **LSPlotsToo.m** – more plotting, used to create TIFF files.
- **MuExplore.m** – calculations of μ and Lagrangian points.
- **MuPlanets.m** – calculations of μ for various planets.
- **showHill.mlapp** – show Hill region, used in S8 Fig.
- **showPhi.mlapp** – show the Kepler Function (generalized Kepler equation), used in Fig 2A.
- **SPF.m** – stereographic projection forward mapping, see Fig 4 and following text.
- **SPG.m** – stereographic projection inverse mapping, see Fig 4 and following text.

S1_Data.zip

Data produced by the MATAB software.

- **CCgridEarthMoon.mat.** Data produced by CCgrid.m and used by CCgridPlot.m.
- **CCgridOneHalf.mat.** Data produced by CCgrid.m and used by CCgridPlot.m.
- **CCgridOneTenth.mat.** Data produced by CCgrid.m and used by CCgridPlot.m.
- **CCgridSunEarth.mat.** Data produced by CCgrid.m and used by CCgridPlot.m.
- **CCgridSunJupiter.mat.** Data produced by CCgrid.m and used by CCgridPlot.m.
- **CCgridSunMars.mat.** Data produced by CCgrid.m and used by CCgridPlot.m.
- **CCgridZero.mat.** Data produced by CCgrid.m and used by CCgridPlot.m.
- **CCminSummary.xlsx** – summary of results of **CCmin.m**.

References

1. Cushman RH, Bates LM. Global aspects of classical integrable systems: Birkhäuser; 2012.
2. Cordani B. The Kepler problem: group theoretical aspects, regularization and quantization, with application to the study of perturbations: Birkhäuser; 2012.
