# Supplementary figures and images for: The symmetry of the Kepler problem, the inverse Ligon-Schaaf mapping and the Birkhoff conjecture
Source: PLoS One. 2018 Sep 13;13(9):e0203821. doi: 10.1371/journal.pone.0203821 (PMC6136791; doi:10.1371/journal.pone.0203821)

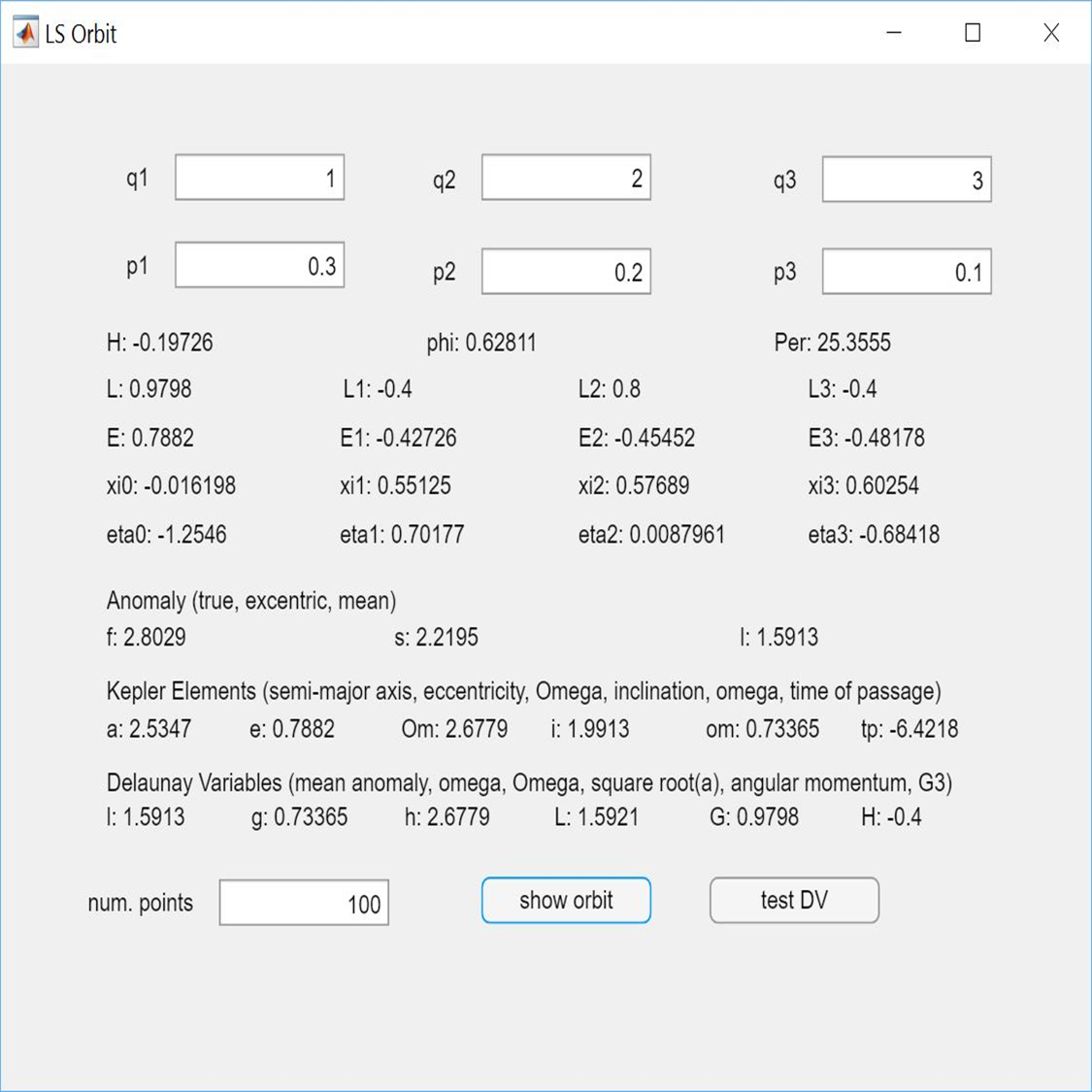

Supplement: S1 Fig — Example Application (software used for Fig 3.) (TIFF) [file pone.0203821.s002.tiff]

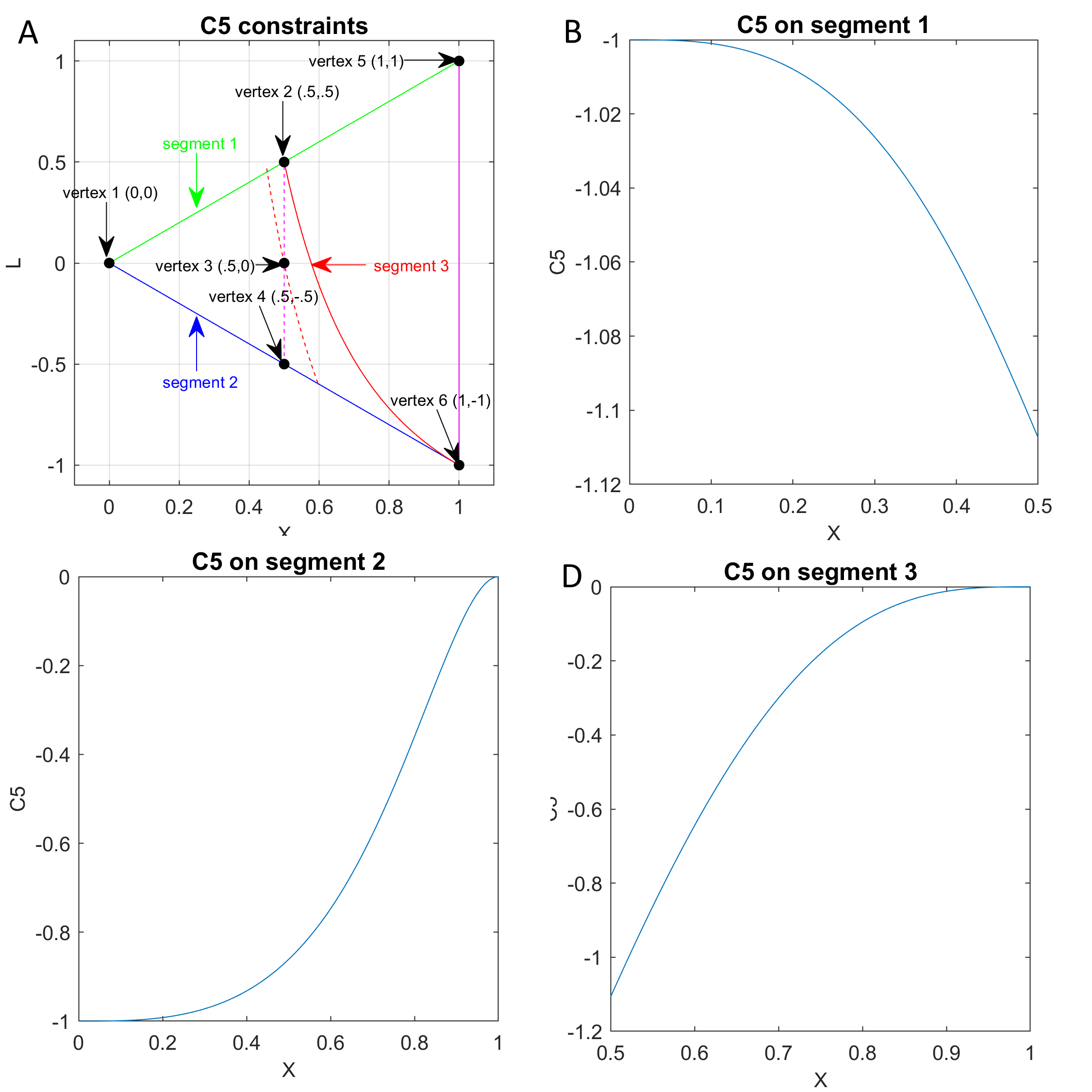

Supplement: S2 Fig — Part of proof of proposition 9. (TIFF) [file pone.0203821.s003.tiff]

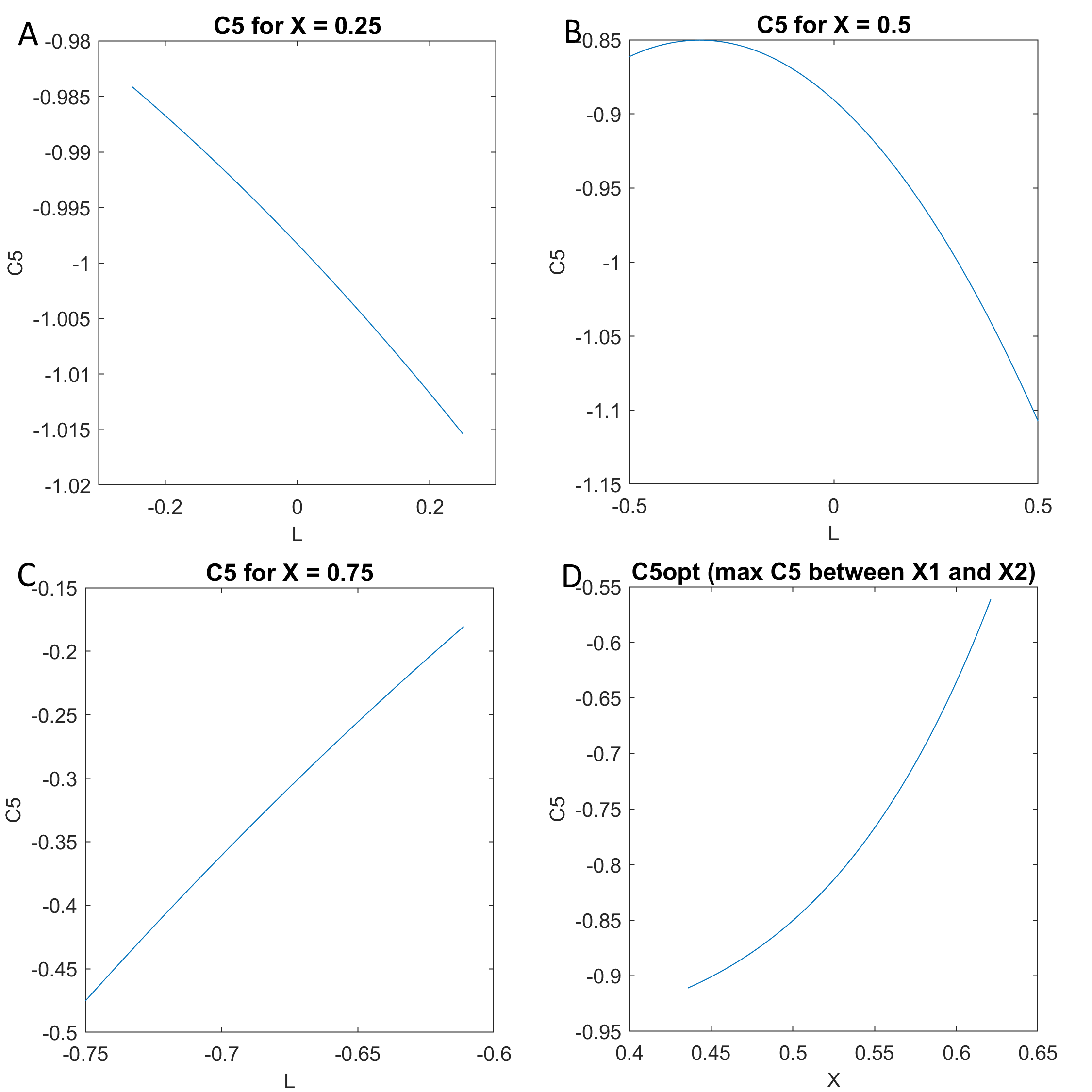

Supplement: S3 Fig — Part of proof of proposition 9. (TIFF) [file pone.0203821.s004.tiff]

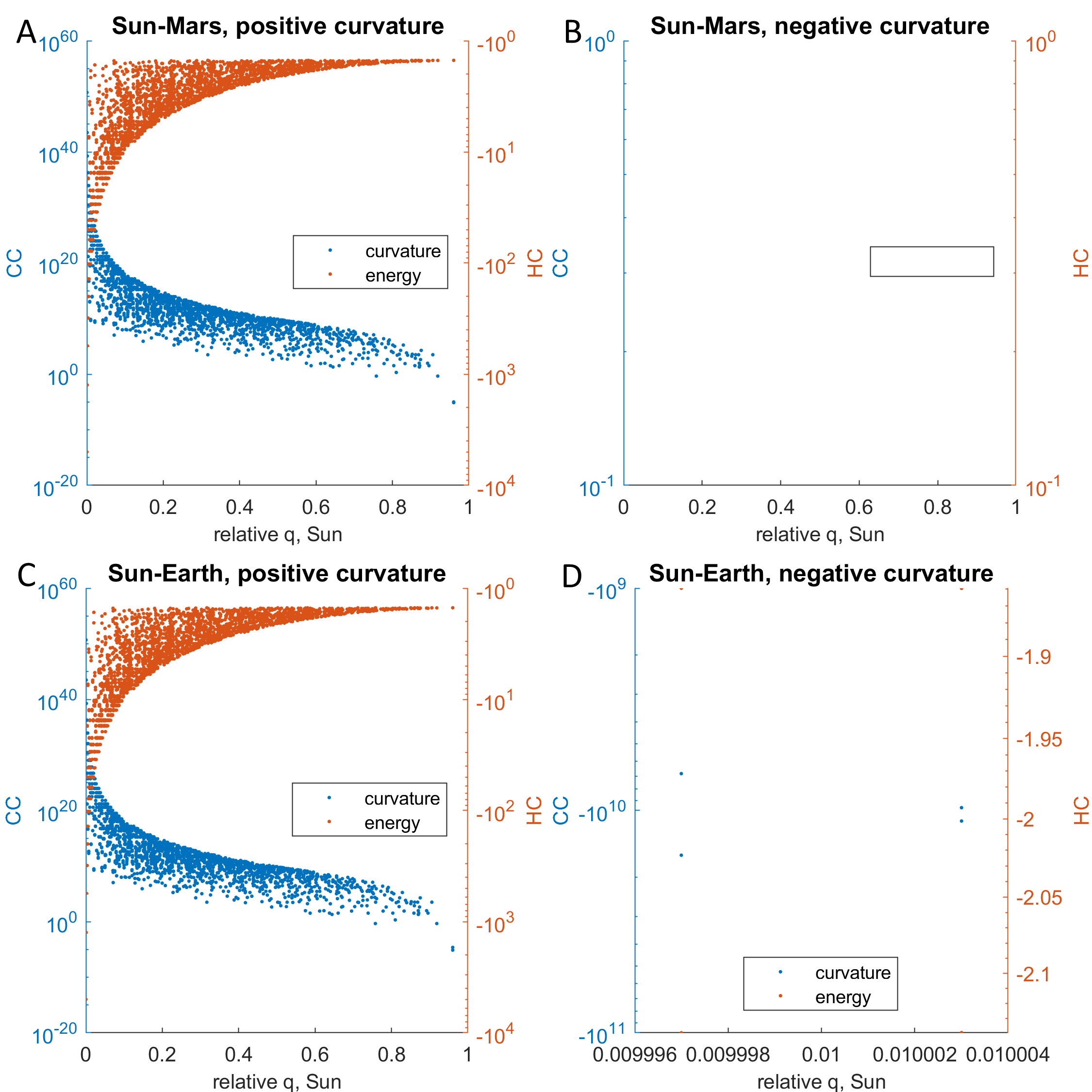

Supplement: S4 Fig — Part of discussion of proposition 10. (TIFF) [file pone.0203821.s005.tiff]

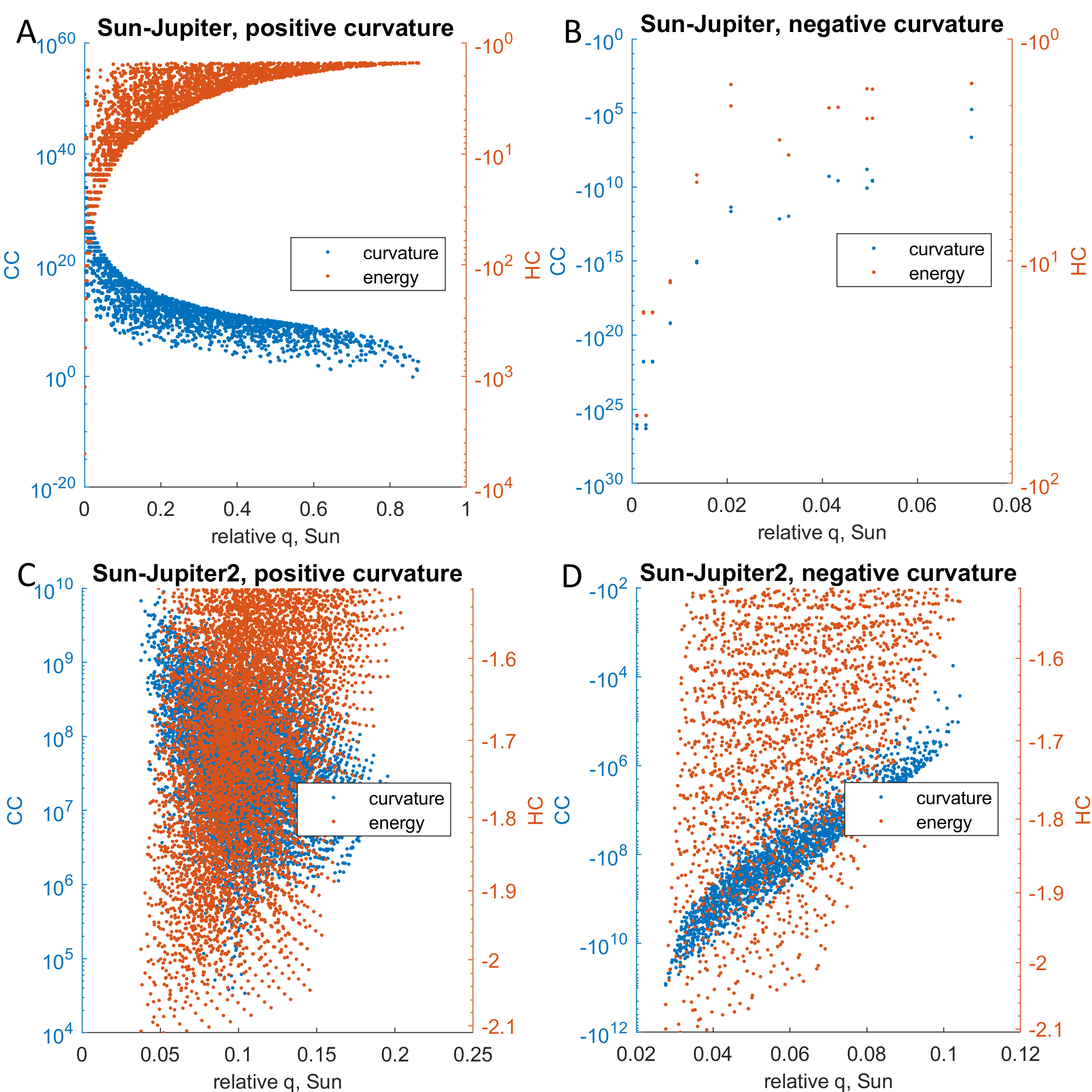

Supplement: S5 Fig — Part of discussion of proposition 10. (TIFF) [file pone.0203821.s006.tiff]

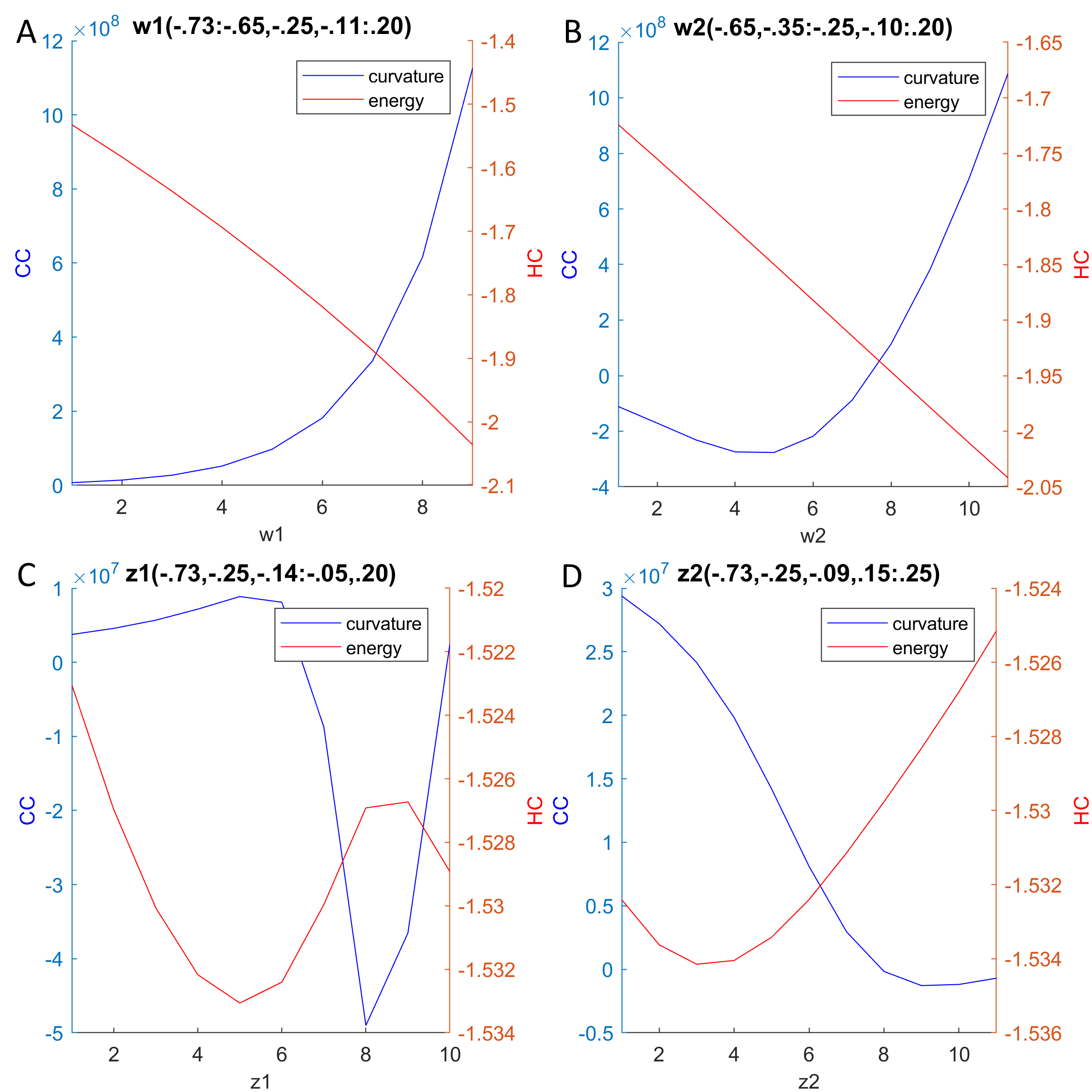

Supplement: S6 Fig — Part of discussion of proposition 10. (TIFF) [file pone.0203821.s007.tiff]

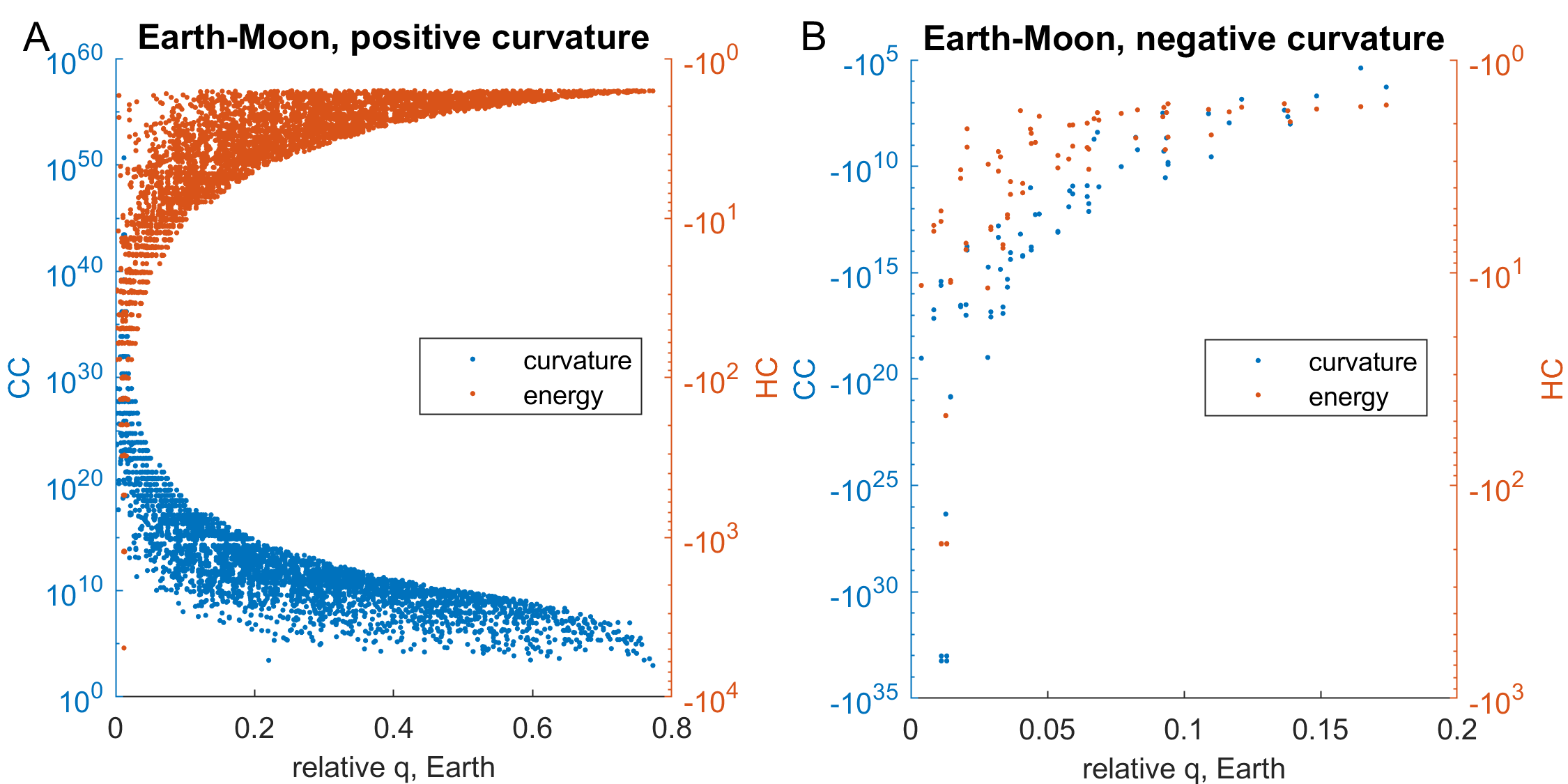

Supplement: S7 Fig — Part of discussion of proposition 10. (TIFF) [file pone.0203821.s008.tiff]

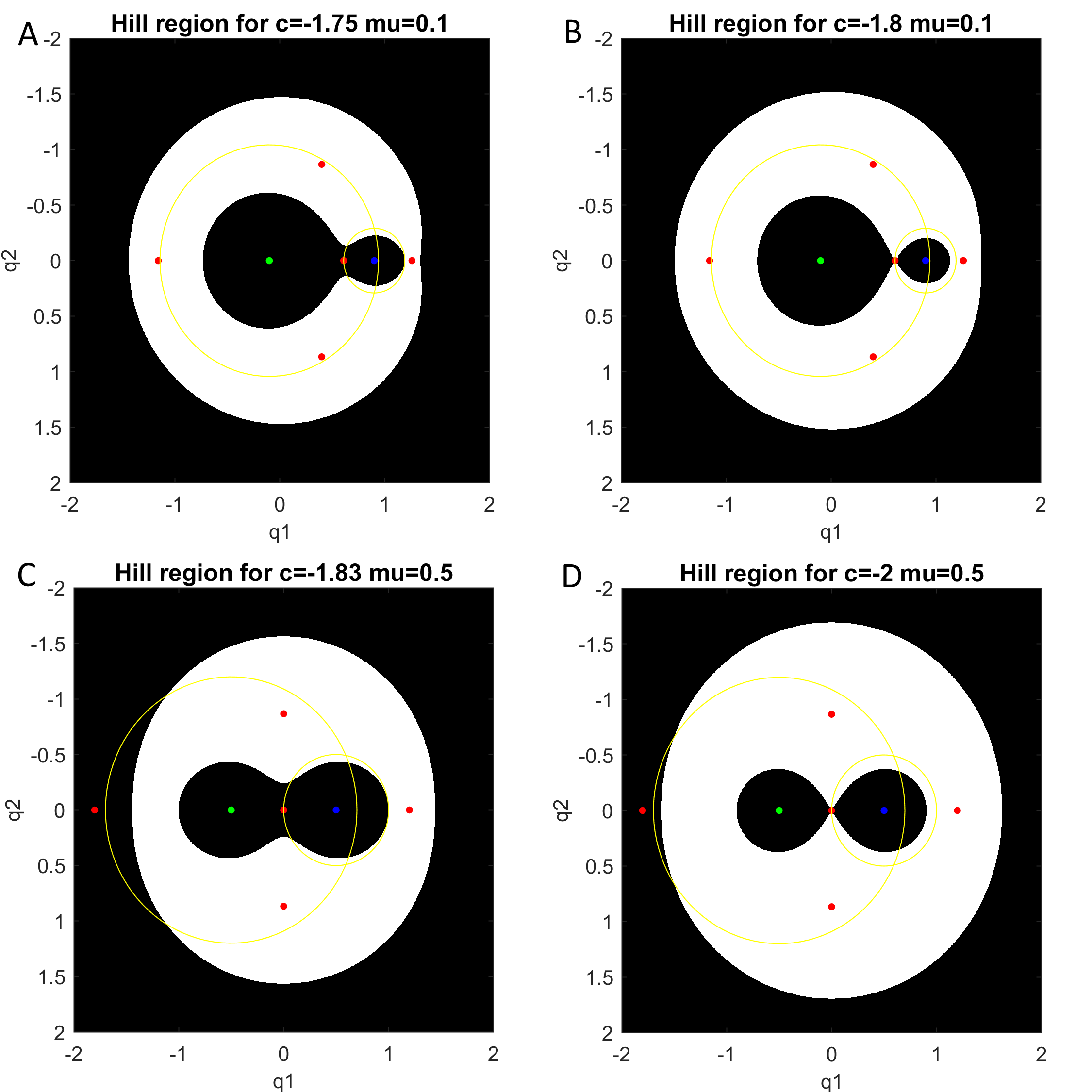

Supplement: S8 Fig — Hill regions for μ = 0.1 and μ = 0.5. Part of discussion of proposition 10. (TIFF) [file pone.0203821.s009.tiff]

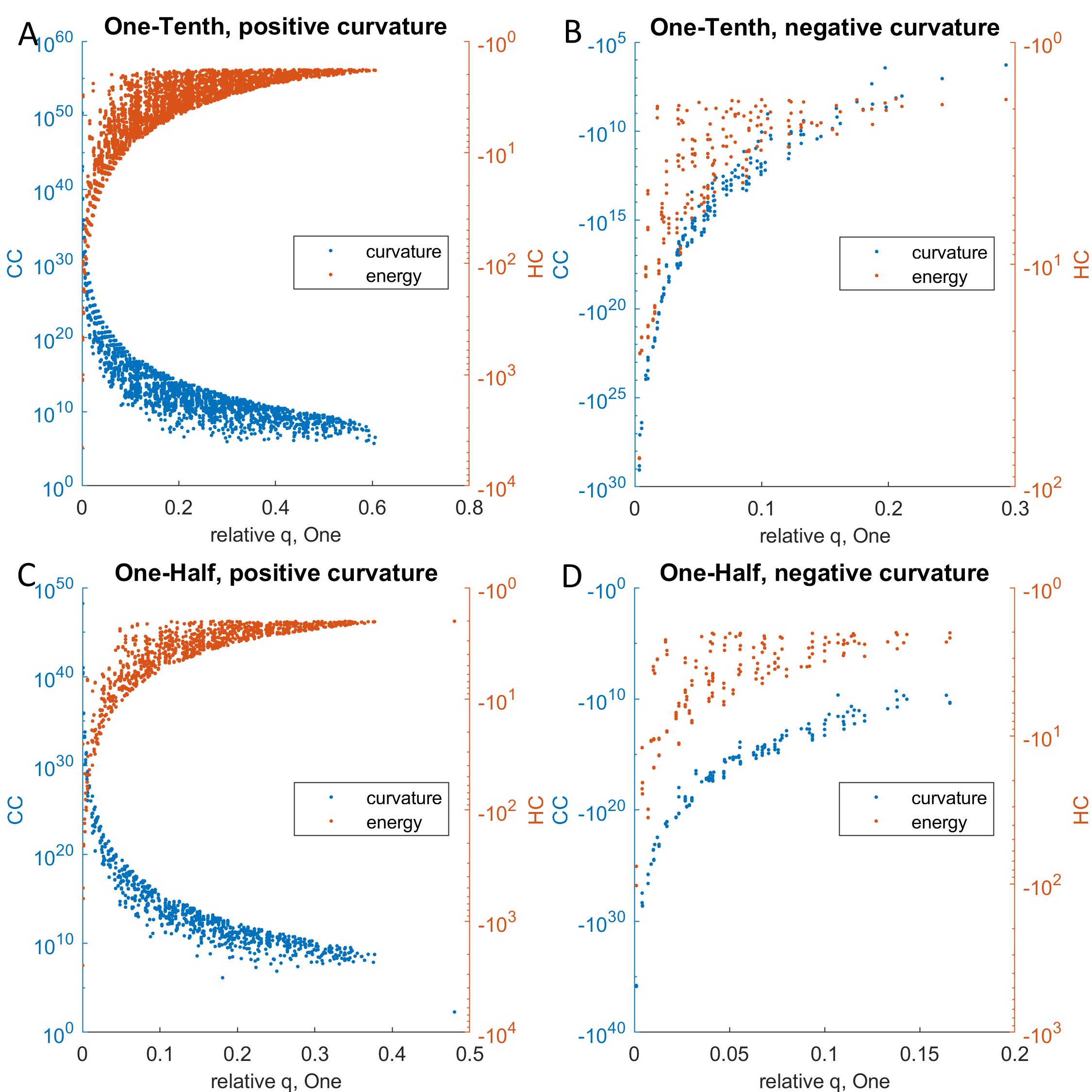

Supplement: S9 Fig — Part of discussion of proposition 10. (TIFF) [file pone.0203821.s010.tiff]
